# Safety and Efficacy of Stem Cell Therapy in Ischemic Stroke: A Comprehensive Systematic Review and Meta-Analysis

**DOI:** 10.3390/jcm14062118

**Published:** 2025-03-20

**Authors:** Abdulrahim Saleh Alrasheed, Tala Abdullah Aljahdali, Israa Aqeel Alghafli, Ghadeer Aqeel Alghafli, Majd Fouad Almuslim, Noor Mohammad AlMohish, Majed Mohammad Alabdali

**Affiliations:** 1 Department of Neurosurgery, College of Medicine, King Faisal University, Al Ahsa 31982, Saudi Arabia; 2 College of Medicine, King Saud bin Abdulaziz University for Health Sciences, Riyadh 11426, Saudi Arabia; aljahdali185@ksau-hs.edu.sa; 3 College of Medicine, King Faisal University, Al Ahsa 31982, Saudi Arabia; 221427343@student.kfu.edu.sa; 4 College of Applied Medical Sciences, Nursing Department, King Faisal University, Al Ahsa 31982, Saudi Arabia; 220026718@student.kfu.edu.sa (G.A.A.); 220011200@student.kfu.edu.sa (M.F.A.); 5 Neurology Department, King Fahad Hospital of the University, Imam Abdulrahman Bin Faisal University, Khobar 31441, Saudi Arabia; nmalmohish@iau.edu.sa; 6 Neurology Department, College of Medicine, Imam Abdulrahman Bin Faisal University, Khobar 31441, Saudi Arabia; mmalabdali@iau.edu.sa

**Keywords:** ischemic stroke, stem cell, therapy, rehabilitation, systematic review, meta-analysis

## Abstract

**Background:** Although recent advancements in ischemic stroke management have reduced associated mortality rates, there remains a pressing need for more reliable, efficacious, and well-tolerated therapeutic approaches due to the narrow therapeutic window of current treatment approaches. The current meta-analysis sought to evaluate the safety and efficacy of stem cell-based therapeutic options for patients with ischemic stroke. **Methods:** PubMed, Web of Science, and Cochrane library databases were searched to retrieve randomized controlled trials (RCTs) evaluating the efficacy and safety of stem cell therapy (SCT) in ischemic stroke patients. Key outcomes included the National Institutes of Health Stroke Scale (NIHSS), modified Rankin Scale (mRS), Barthel Index (BI), Fugl–Meyer Assessment (FMA), infarct size, and safety profile. The random effects model with the continuous method was used to calculate the pooled effect size in Review Manager 5.4.1, and subgroup analyses were performed based on demographics, stroke duration, and SCT delivery protocols. **Results:** A total of 18 RCTs involving 1026 patients were analyzed, with 538 in the treatment group and 488 in the control group. The mean change in NIHSS score was comparable between groups [MD = −0.80; 95% CI: −2.25, 0.65, *p* < 0.0001]. However, SCT showed better outcomes in mRS [MD = −0.56; 95% CI: −0.76, −0.35, *p* = 0.30] and BI scores [MD = 12.00; 95% CI: 4.00, 20.00, *p* = 0.007]. Additionally, the mean change in FMA score was significantly greater with SCT [MD = 18.16; 95% CI: 6.58, 29.75, *p* = 0.03]. The mean change in infarct volume also favored stem cell therapy [MD = 8.89; 95% CI: −5.34, 23.12, *p* = 0.08]. The safety profile was favorable, with adverse event rates comparable to or lower than controls. **Conclusions:** SCT offers a safe and effective approach to improving functional outcomes in stroke patients, particularly with early intervention. These findings highlight the potential of SCT in ischemic stroke rehabilitation while underscoring the need for standardized protocols and long-term safety evaluation.

## 1. Introduction

Cerebrovascular accident (CVA), commonly known as stroke, which results from cerebral ischemia and hypoxia or hemorrhage, is the global leading cause of both disability and mortality [1,2]. The most common type of stroke is ischemic stroke, which accounted for 62.4% of all stroke incidents globally in 2019 [3]. Previous reports indicate that ischemic stroke leads to a mortality rate of up to 15% within 30 days, with up to 50% of survivors experiencing long-term disability. Additionally, over 40% of stroke survivors suffer a subsequent ischemic stroke, with a significantly increased likelihood of death or severe disability [4].

Current therapeutic approaches focus on maximizing cerebral perfusion in the ischemic penumbra and preventing or reversing brain damage. Anticoagulants and thrombolytics play a key role in restoring nervous system function [5,6,7]. The primary limitation of this therapy is the narrow window of administration, which must be within 4.5 h from symptom onset. Patients who are ineligible for thrombolytic therapy or thrombectomy face substantially higher rates of disability. Although rehabilitation therapy supports the recovery of neurological function, its overall therapeutic effectiveness remains limited [4]. 

Even though numerous studies have been conducted to enhance the brain’s ability to prevent ischemic stroke, the brain’s inherent capacity for recovery following a stroke remains limited. Two promising therapeutic approaches include activating the brain’s natural defense mechanisms, introducing external stem cells, and progenitor cells. In this context, stem cell therapy (SCT) has emerged as a novel therapeutic strategy for ischemic stroke management [8,9,10]. A growing body of evidence from preclinical studies and clinical trials suggests that stem cell administration can modulate the multiple pathways involved in stroke recovery [8,11]. 

Stem cells, including mesenchymal, hematopoietic, and neural stem cells, are being used in regenerative medicine as a potentially effective way to treat stroke-related tissue damage, encourage tissue repair, and improve functional recovery [11,12]. In 2005, five stroke patients received the first autologous mesenchymal stem cell (MSC) transplants, showing improved functional recovery after one year without any cell-related side effects [13]. Since then, numerous clinical trials have explored different stem cell types, dosages, and delivery methods at various stages of ischemic stroke to assess the safety and efficacy of stem cell-based therapies for those patients. However, the outcomes across various stroke assessment scales have been inconsistent [14,15]. Furthermore, the safety of this treatment and its potential to aid in ischemic stroke rehabilitation remain crucial questions. In this meta-analysis, we aim to assess the efficacy and safety of stem cell treatment for ischemic stroke.

## 2. Methods

To ensure methodological rigor, the Preferred Reporting Items for Systematic Reviews and Meta-Analyses (PRISMA) guidelines were followed to conduct the current meta-analysis [16]. Prospectively, we registered our meta-analysis in the International Prospective Register of Systematic Reviews (PROSPERO) (ID CRD42024556509).

### 2.1. Search Strategy

Two researchers independently searched the PubMed, Web of Science, and Cochrane library databases through June 2024 for randomized controlled trials (RCTs) assessing the efficacy and safety of SCT in ischemic stroke patients. Keywords used in the research strategy included Medical Subject Headings (MeSH) and Entry Terms. The keywords used in this search strategy included (“Stem Cell” OR “Cell Transplantation”) AND (“Stroke” OR “Ischemic Stroke” OR “Ischemic Brain” OR “Cerebrovascular Accident” OR “CVA” OR “Brain Infarction”) AND (“Efficacy” OR “Safety” OR “Tolerability” OR “Outcome” OR “Impact” OR “Effect” OR “mRS” OR “NIHSS” OR “BI”). 

### 2.2. Inclusion and Exclusion Criteria

The inclusion criteria for eligible studies were as follows: (1) patients diagnosed with ischemic stroke aged 18 years or older; (2) treatment involving stem cell-based therapeutic interventions, regardless of the type, dose, or mode of delivery; (3) control groups including standard care, other therapeutic interventions for ischemic stroke, or placebo; (4) assessment of efficacy and/or safety outcomes, including the National Institutes of Health Stroke Scale (NIHSS), modified Rankin Scale (mRS), Barthel Index (BI), Fugl–Meyer Assessment (FMA), infarct volume, and safety outcomes such as overall adverse events (AEs), serious adverse events (SAEs), immediate adverse events, and delayed adverse events; (5) studies published between inception and June 2024; and (6) English-written randomized controlled trials (RCTs). 

The exclusion criteria were as follows: (1) interventions other than stem cells-based interventions; (2) studies addressing diseases other than ischemic stroke; (3) study designs other than English-written RCTs; (4) populations including children, patients with hemorrhagic stroke, or those with pre-existing significant disabilities; or (5) outcome measures not aligning with the predefined measures.

## 3. Study Selection

Initial search results were uploaded to Rayyan Software 5.2 for selection, screening, and duplicate removal. Relevant papers found through the database searches were screened by title and abstract by two independent reviewers to assess the potential eligibility [17]. Potential eligible studies were subsequently subjected to full-text review by two independent review teams. Any disagreements that arose were resolved through consultation with a third reviewer.

### Data Extraction

Four independent reviewers meticulously extracted relevant data from the included studies and systematically organized them into a comprehensive spreadsheet. The extracted data covered key information such as the first author’s name, publication year, study design, country of origin, and sample size. Additionally, patient demographics, including age, BMI, gender, race, and ethnicity, were recorded. Details of the stem cell interventions were also captured, including the tissue source, intervention regimen (e.g., anesthetic used, cell dosage, method of administration, timing of injections), and follow-up periods.

## 4. Statistical Analysis

Mean differences (MDs) were calculated for the mean change (baseline versus the end of the follow-up period) for the NIHSS score, mRS score, BI, and FMA score. The random effects model with the continuous method was used to calculate the pooled effect size in Review Manager 5.4 [18]. Moreover, a random effects model with the dichotomous method was used to calculate the pooled safety outcome of the included studies. The results obtained from the pooled studies were demonstrated in forest plots. The results obtained from the pooled studies were demonstrated in forest plots, and a funnel plot was formed for all the primary outcomes to assess the publication bias. Higgin’s I square test was used to assess heterogeneity levels (low < 25 percent, moderate 25–75 percent, high > 75 percent) [19]. Analysis was deemed significant when the *p*-value was less than 0.05.

## 5. Heterogeneity Exploration

To explore the effect of clinical heterogeneity between the studies on meta-analysis outcomes, subgroup analyses were performed based on the region where the original studies were conducted, the age distribution, the gender distribution, the patient count, the duration of stroke, and the duration of follow-up. Sensitivity analysis assessed each study’s contribution to the pooled estimate by excluding individual trials one at a time and recalculating the pooled estimate for the remaining studies (leave-one-out meta-analysis) [20].

## 6. Results

### 6.1. Search Results

**Figure 1:**
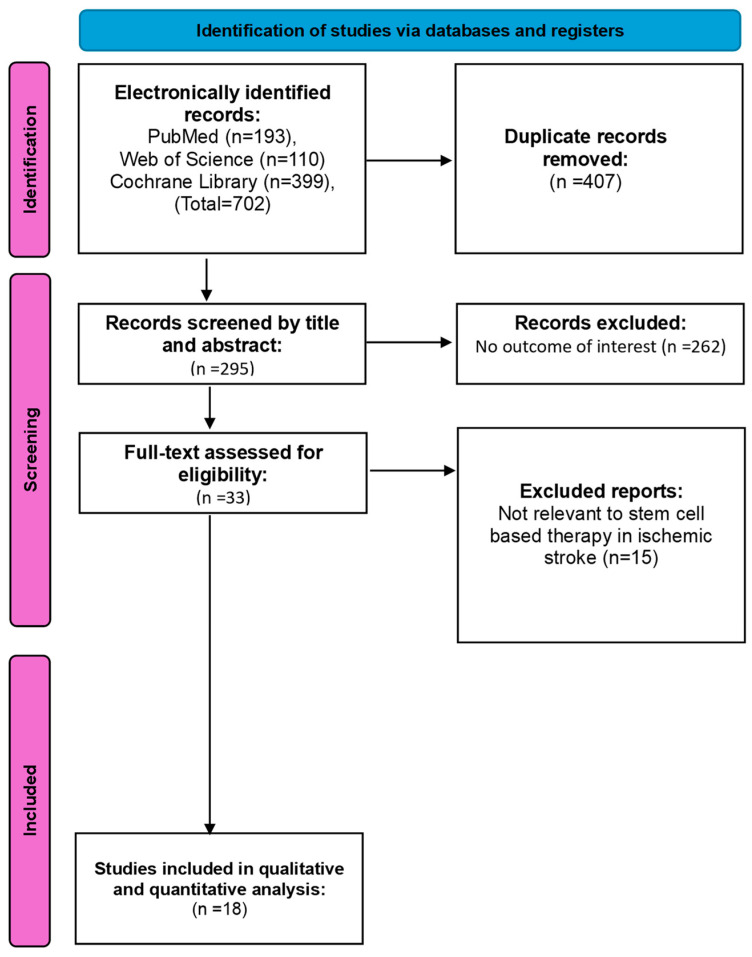
PRISMA flowchart of the identification of the studies.

A flowchart illustrating the study selection process is presented in (Figure 1). The initial search yielded 702 records, of which 407 were identified as duplicates and subsequently excluded. This left 295 unique articles for title and abstract screening. Among these, 262 articles were excluded for failing to meet the eligibility criteria. The remaining 33 full-text articles underwent detailed evaluation. Of these, 15 articles were excluded as they did not address relevant outcomes of interest. In the end, 18 RCTs were deemed eligible and included in the meta-analysis.

### 6.2. Study Characteristics

(Table 1 and Table 2) presents the baseline characteristics of the studies investigating the impact of SCT on ischemic stroke patients. We highlighted the key demographic and clinical details across 18 RCTs conducted from 2005 to 2024 in various countries, including South Korea, India, Spain, China, USA, UK, Japan, France, and Malaysia.

The total patient sample sizes varied significantly across the studies, with SCT-treated patient groups ranging from 4 [21] to 104 [22] participants and control groups ranging from 6 [23] to 102 [22] participants. Gender distribution showed variability, with some studies, such as the study conducted by Law et al. in 2021 [24], reporting a predominantly male SCT-treated group (88.9%), while other studies, such as Celis-Ruiz et al. (2022) [21], had a higher proportion of female participants (75%). The studies also reported vast differences in age, stroke duration, and follow-up periods. The mean age ranged from 48.4 years [25] to 79.25 years [21], while the duration after stroke varied from acute phases (e.g., 18–36 h [22]) to chronic conditions, e.g., 31 months [26]. Follow-up periods extended from as short as 32.7 days [27] to as long as 4 years [23], providing a broad range of post-treatment assessments.

Clinical baseline indicators such as infarct volume, NIHSS scores, and BI also showed considerable variation. For instance, baseline NIHSS scores, an indicator of stroke severity, ranged from 5 in Wang et al. 2020 [26] to 17 in Law et al. 2021 [24], while baseline BI scores, reflecting functional independence, fluctuated across studies, with higher baseline values reported as 76.3 [28].

**Table 1:** Baseline characteristics of the included studies.

Study (Author)	Year of the Study	Country of the Study	Cell Type	Dosage	Administration	Administration Timing	Total Patients, N		Gender Distribution of SCT Group (%)	
							SCT Group	Control Group	Males	Females
Bang et al. [13]	2005	South Korea	Mesenchymal stem cell	5 × 10^7^, twice	IV	32–61 days	5	25	80	20
Lee et al. [29]	2010	South Korea	Mesenchymal stem cell	5 × 10^7^, twice	IV	4 to 9 weeks	16	36	50	50
Chen et al. [30]	2014	China	Peripheral blood stem cell	3–8 × 10^6^	Stereotaxic implantation	6 months to 5 years	15	15	80	20
Prasad et al. [31]	2014	Indian	Mesenchymal stem cell	Mean of 2.8 × 10^8^	IV	Mean of 18.5 days	60	60	68.3	60
Bhasin et al. [25]	2016	India	Bone marrow-derived mononuclear cell	Mean of 6.28 × 10^7^	IV	3 months to 2 years	20	20	75	25
Hess et al. [32]	2017	USA and UK	Multipotent adult progenitor cell	1.2 × 10^9^	IV	24 to 48 h	71	63	56.3	43.7
Bhatia et al. [28]	2018	India	Bone marrow-derived mononuclear cell	Mean of 6.1 × 10^8^	IA	Mean of 10 days	10	10	80	20
Fang et al. [23]	2019	China	Endothelial progenitor cell, (50%); mesenchymal stem cell (50%)	2.5 × 10^6^/kg, twice	IV	Mean of 33.5 days	5	6	80	20
Savitz et al. [33]	2019	America	Bone marrow-derived ALDHbr cells (ALD-401)	Mean of 3.08 × 10^6^	IA	13 to 19 days	29	19	69	31
Wang et al. [26]	2020	China	Olfactory ensheathing cell	10 × 10^6^	Intranasal	>12 months	18	9	83.2	16.7
Jaillard et al. [34]	2020	France	Mesenchymal stem cell	10 × 10^7^ (First cohort), 30 × 10^7^ (Second cohort)	IV	<5–6 weeks	16	15	68.8	31.2
Law et al. [24]	2021	Malaysia	Bone marrow-derived mononuclear cell	2 × 10^6^/kg	IV	Median of 63 days	9	8	88.9	11.1
Chung et al. [27]	2021	South Korea	Mesenchymal stem cell	1 × 10^6^/kg	IV	>3 months	39	15	43.6	56.4
Celis-Ruiz et al. [21]	2022	Spain	Mesenchymal stem cell	1 × 10^7^	IV	Mean of 13 days	4	8	25	75
Lee et al. [35]	2022	South Korea	Mesenchymal stem cell	11 × 10^6^	IV	>3 months	31	13	48.3	51.7
Moniche et al. [36]	2023	Spain	Bone marrow-derived mononuclear cell	(2 × 10^6^/kg or 5 × 10^6^/kg)	IA	Median of 3 days	39	38	54	56
Houkin et al. [22]	2024	Japan	Multipotent adult progenitor cell	1.2 × 10^9^	IV	18 to 36 h	104	102	53.8	46.2
Laskowitz et al. [37]	2024	USA	Umbilical cord blood	0.5–5 × 10^7^ total nucleated cell count/kg	IV	3–10 days	47	26	61.7	38.3

SCT: Stem cell therapy; N/A: Not applicable; IV: Intravenous; IA: Intra-arterial; Bone mar-row-derived; ALDHbr: aldehyde dehydrogenase.

**Table 2:** Baseline characteristics of the included studies.

Study (Author)	Age Distribution, Mean (SD)		Duration After Stroke, Mean (SD)		Duration of Follow-Up, Mean (SD)		Baseline Infarct Volume, Mean (SD)		Baseline NIHSS Score, Mean (SD)		Baseline BI, Mean (SD)	
	SCT Group	Control Group	SCT Group	Control Group	SCT Group	Control Group	SCT Group	Control Group	SCT Group	Control Group	SCT Group	Control Group
Bang et al. [13]	63 (7.5)	59.3 (11.5)	7 days	7 days	12 months	12 months	127.4 (70.3)	89.1 (77.4)	10.6 (2.6)	11.6 (4.9)	9.0 (20.1)	13.4 (22.2)
Lee et al. [29]	64.0 (11.6)	64.9 (14.5)	7 days	7 days	129.6 weeks	110.3 weeks	115.7 (95.2)	90.1 (86.8)	10.63 (3.03)	10.17 (3.67)	N/A	N/A
Chen et al. [30]	50.1 (7.7)	52.8 (9.0)	2.7 years	2.5 years	12 months	12 months	N/A	N/A	9.3 (0.5)	9.6 (1.3)	N/A	N/A
Prasad et al. [31]	50.7 (11)	52.5 (12)	17 days	17 days	6 months	6 months	86.9 (57)	111.7 (72.4)	11 (3)	13 (3)	25 (12.7)	27.5 (11)
Bhasin et al. [25]	48.4 (8.16)	49.6 (5.6)	11.05 months	10.5 months	56 days	56 days	N/A	N/A	N/A	N/A	46.5 (5.9)	N/A
Hess et al. [32]	61.8 (11.4)	62.6 (11.4)	37.2 h	39.3 h	12 months	12 months	43.7 (26.9)	50.9 (41.3)	13.4 (3.6)	13.3 (3,7)	N/A	N/A
Bhatia et al. [28]	57 (12.2)	66 (7.3)	10 days	10 days	6 months	6 months	N/A	N/A	10.6	10.5	76.3	78.1
Fang et al. [23]	50.1 (7.55)	52.83 (14.95)	7 days	7 days	4 years	4 years	N/A	N/A	12.20 (4.92)	15.5 (3.02)	39.00 (24.60)	25 (20)
Savitz et al. [33]	59.3 (10.03)	62.9 (10.81)	28 days	16 days	90 days	90 days	N/A	N/A	11	10	N/A	N/A
Wang et al. [26]	64.2 (5.7)	66 (12)	6 days	N/A	6 months	6 months	69 (23.9)	79 (33.5)	12.5 (1.4)	12.3 (4.5)	N/A	N/A
Jaillard et al. [34]	55 (6)	53 (9.12)	<14 days	<14 days	2 years	2 years	92 (60.1)	113 (48.2)	17 (3.48)	17 (3.33)	48.75 (22.5)	45 (20)
Law et al. [24]	55 (6)	53 (9.12)	<14 days	<14 days	2 years	2 years	92 (60.1)	113 (48.2)	17 (3.48)	17 (3.33)	48.75 (22.5)	45 (20)
Chung et al. [27]	63.03 (14.36)	64.27 (13.25)	21 days	18.4 days	32.7 days	30.6 days	90.96 (79.57)	96.46 (74.31)	11.36 (5.2)	14.5 (5.32)	28.28 (26.63)	19.8 (25.5)
Celis-Ruiz et al. [21]	79.25 (3.83)	77.13 (3.75)	13.4 days	12.5 days	2 years	2 years	43.22 (41.84)	88.16 (56.15)	10.5 (3.19)	11 (1.5)	N/A	N/A
Lee et al. [35]	63.4 (14.0)	61.5 (13.0)	24.6 days	20.9 days	90 days	90 days	125 (115.7)	127.3 (122.7)	N/A	N/A	N/A	N/A
Moniche et al. [36]	64.2 (5.7)	66 (12)	6 days	N/A	6 months	6 months	69 (23.9)	79 (33.5)	12.5 (1.4)	12.3 (4.5)	N/A	N/A
Houkin et al. [22]	76.7 (10.4)	76.2 (10.6)	18–36 h	18–36 h	12 months	12 months	42.0 (48.4)	54.3 (57.0)	13.7 (3.9)	13.9 (3.9)	N/A	N/A
Laskowitz et al. [37]	62.6 (12.1)	64.4 (11.2)	6 days	6 days	90 days	90 days	N/A	N/A	12.3 (3.6)	12.2 (3.4)	40 (9.4)	45

SCT: Stem cell therapy; N/A: Not applicable; NIHSS: National Institutes of Health Stroke Scale; BI: Barthel Index.

### 6.3. Risk of Bias Assessment

**Figure 2:**
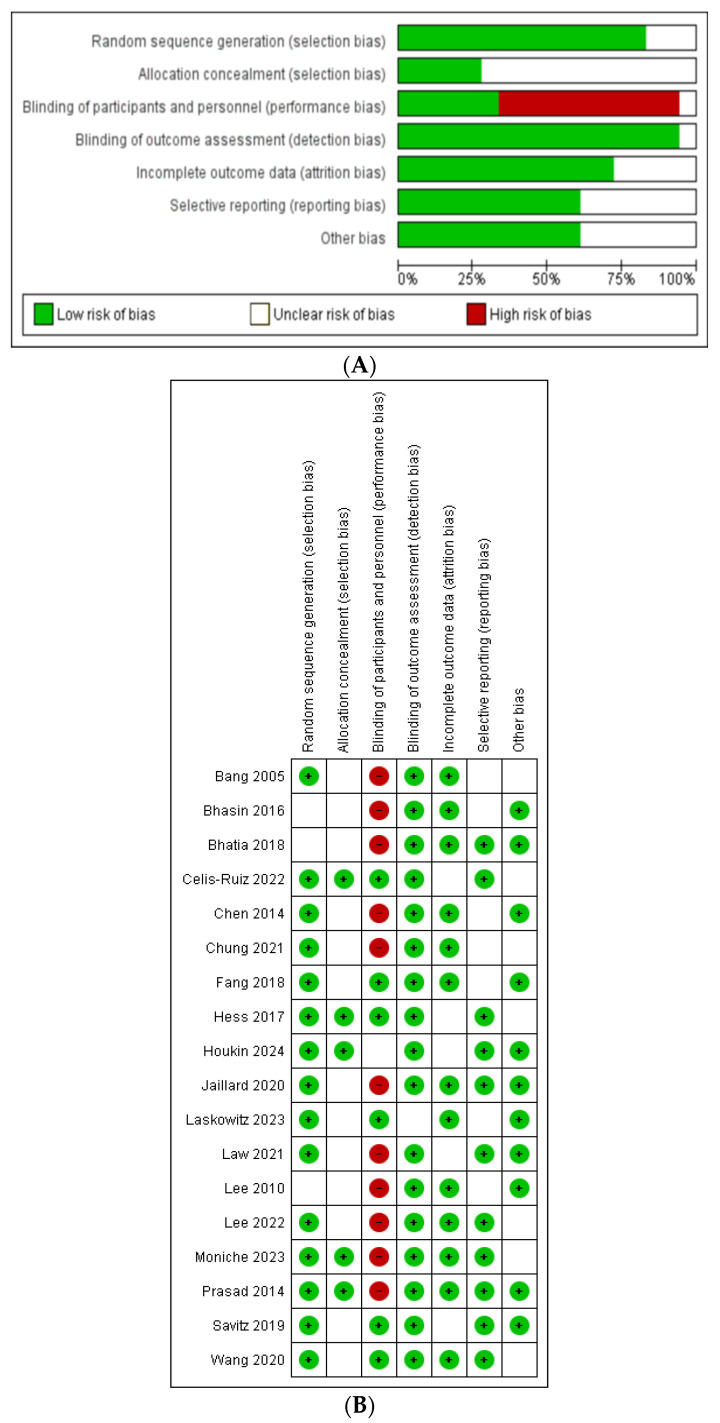
Risk of bias graph (**A**) and summary (**B**) of the quality assessment of the included studies [13,21,22,23,24,25,26,27,28,29,30,31,32,33,34,35,36,37].

We used the Cochrane risk of bias (RoB 2.0) tool for the quality assessment of the studies [38]. The findings were as follows: random sequence generation and blinding of outcome assessment showed a low bias risk. Allocation concealment and selective reporting displayed a small amount of unclear or high risk of bias. Blinding of participants and personnel exhibited the highest proportion of high risk of bias. Incomplete outcome data indicated a low primary risk but with some unclear risk. Other biases had minimal high risk but were generally low risk. The risk of bias summary and graph are given in Figure 2 below.

### 6.4. Publication Bias

The funnel plots for the primary outcomes, including the comparison of mean change in the NIHSS score, mRS score and Barthel Index, FMA score, infarct volume, and safety outcomes of the pooled studies, were symmetric and suggested no obvious publication bias in the included studies’ reporting of the patient outcomes (Supplementary Figures S1–S6).

## 7. Evaluation of the Efficacy Outcomes

### 7.1. Difference in the Mean Change in NIHSS Score

**Figure 3:**
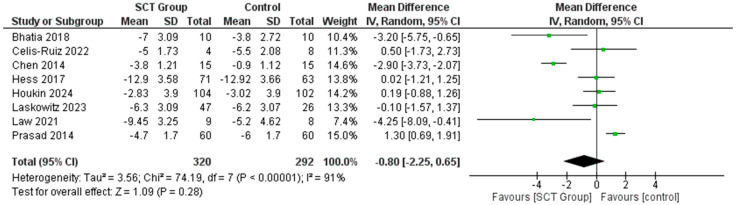
Forest plot for the pooled analysis of difference in the mean change in NIHSS score [21,22,24,28,30,31,32,37].

Our meta-analysis demonstrated that the mean change in NIHSS score was comparable in both SCT and control groups in the included studies [Mean Difference MD = −0.80; 95% CI: −2.25, 0.65, *p* < 0.0001] (Figure 3). Sensitivity analysis was performed using the leave-one-out method, which did not show any significant change in the pooled results when removing just one of the studies (Supplementary File S1; Figure S7).

### 7.2. Difference in the Mean Change in mRS Score

**Figure 4:**
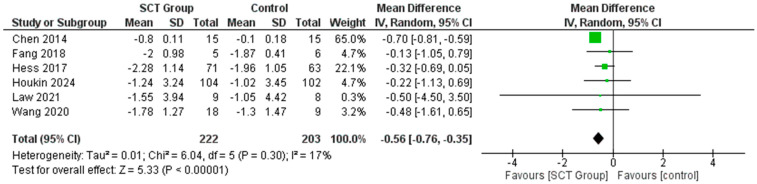
Forest plot for the pooled analysis of the difference in the mean change in mRS score [22,23,24,26,30,32].

Our meta-analysis demonstrated that the mean change in mRS score was greater in the SCT group than in the control groups in the included studies [Mean Difference MD = −0.56; 95% CI: −0.76, −0.35, *p* = 0.30] (Figure 4). Sensitivity analysis was performed using the leave-one-out method, which did not show any significant change in the pooled results when removing just one of the studies (Supplementary Figure S8).

### 7.3. Difference in the Mean Change in BI

**Figure 5:**
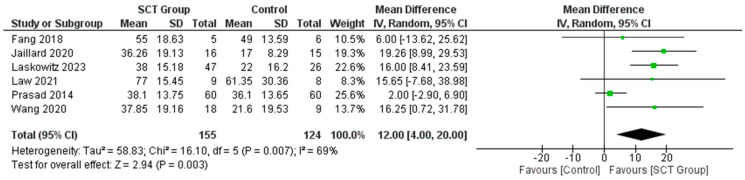
Forest plot for the pooled analysis of the difference in the mean change in BI [23,24,26,30,31,34].

Our meta-analysis demonstrates that the mean change or mean increase in the BI was significantly greater in the SCT as compared to the control groups in the included studies [Mean Difference MD = 12.00; 95% CI: 4.00, 20.00, *p* = 0.007] (Figure 5). Sensitivity analysis was performed using the leave-one-out method, which did not show any significant change in the pooled results when removing just one of the studies (Supplementary Figure S9).

### 7.4. Difference in the Mean Change in FMA Score

**Figure 6:**
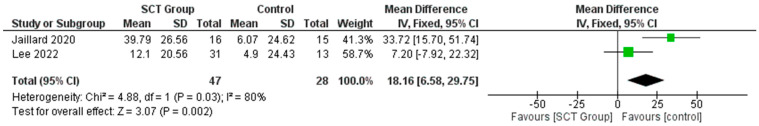
Forest plot for the pooled analysis of the difference in the mean change in FMA score [29,34].

Our meta-analysis demonstrated that the mean change in FMA score was significantly greater in the SCT than in the control groups in the included studies [Mean Difference MD = 18.16;95% CI: 6.58, 29.75, *p* = 0.03] (Figure 6).

### 7.5. Difference in the Mean Change in Infarct Volume

**Figure 7:**
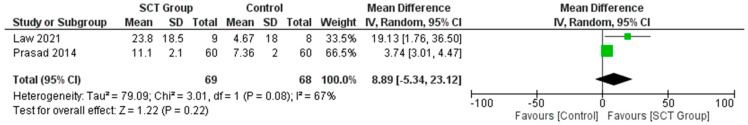
Forest plot for the pooled analysis of the difference in the mean change in infarct volume [24,31].

Our meta-analysis demonstrated that the mean change in infarct volume was greater in the SCT as compared to the control groups in the included studies [Mean Difference MD = 8.89; 95% CI: −5.34, 23.12, *p* = 0.08] (Figure 7).

## 8. Safety Outcomes

Table 3 displays a comprehensive analysis of the safety outcomes in the multiple studies of SCT among ischemic stroke patients. It reveals a favorable safety profile, though with notable variations between different trials. Of the 18 studies assessed, approximately half (9 studies) reported that neither the treatment nor the control groups showed any serious adverse effects, pointing to a promising level of safety for the intervention. The study by Hess et al. (2017) [32], the largest to date, found comparable rates of adverse events between groups (34% in SCT, 39% in control), providing solid evidence supporting the treatment’s relative safety. Others found markedly lower rates of complications in their SCT group compared to the control group. de Celis-Ruiz et al. (2022) [21] noticed that the incidence of serious adverse effects among SCT patients compared to the control group was 0% to 44.4%, and a similar pattern was echoed by Lee et al. (2010) [29] with a percentage of 75% to 80.5%, suggesting protective effects of the therapy. 

Nonetheless, a few studies presented opposing trends. The study by Prasad et al. (2014) [31] observed higher rates of serious adverse reactions in the group that received treatment (20%) compared to the controls (13.3%), which is noteworthy. The incidence rates of serious adverse events varied considerably across studies. They ranged between 0% and 75% for groups subjected to SCT and between 0% and 80.5% for control groups. This statistic underlines the potential impact of different treatment procedures, patient populations, and reporting criteria in other studies. The overall pattern, however, even in such conditions, points to the fact that SCT has a good safety profile, with the most common adverse events being mild fever, headache, and fatigue, typically resolving without long-term consequences. Serious adverse events, such as infections, seizures, and nausea, were rare and varied widely across studies (0–41%), with higher rates often linked to procedural variability or patient-specific factors. In most studies, most adverse event rates were comparable or even lower than those observed in the control groups. Almost all the studies reported favorable immediate adverse effects profiles, though the incidence varied widely across studies (0–99%). In contrast, delayed adverse effects were consistently reported; however, their documentation was not clearly linked to SCT due to inconsistencies in the investigative methods and the formulation of their association with the treatment.

### 8.1. Serious Adverse Effects

**Figure 8:**
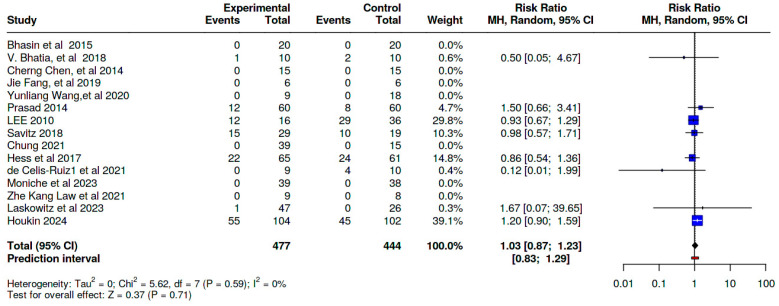
A pooled analysis of the serious adverse effects profiles of the included studies [21,22,23,24,25,26,27,28,29,30,31,32,33,36,37].

Our meta-analysis demonstrates that the incidence of serious adverse effects in the SCT groups was comparable to that in the control groups across the included studies, with no statistically significant difference [Risk Ratio (RR) = 1.03; 95% CI: 0.87, 1.23; *p* = 0.59] (Figure 8; Figure S10).

### 8.2. Immediate Adverse Effects

**Figure 9:**
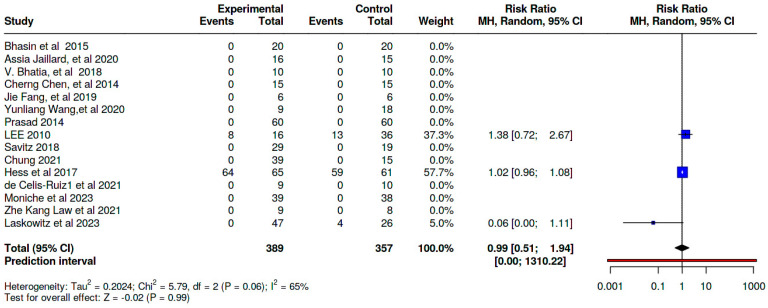
A pooled analysis of the immediate adverse effects profiles of the included studies [21,23,24,25,26,27,28,29,30,31,32,33,34,36,37].

Our meta-analysis demonstrates that the incidence of immediate adverse effects in the SCT groups was comparable to that in the control groups across the included studies, with no statistically significant difference [Risk Ratio (RR) = 0.99; 95% CI: 0.51,1.94; *p* = 0.06] (Figure 9; Figure S11).

### 8.3. Delayed Adverse Effects

**Figure 10:**
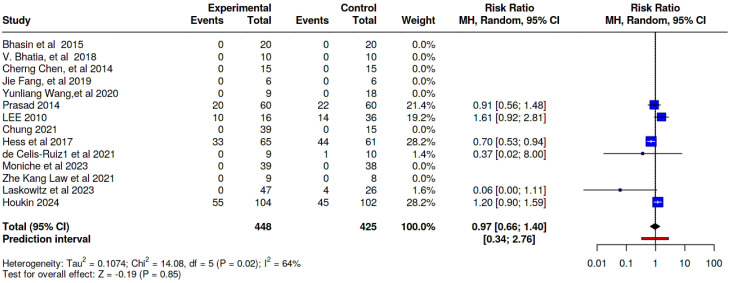
A pooled analysis of the delayed adverse effects profile of the included studies [21,22,23,24,25,26,27,28,29,30,31,32,36,37].

Our meta-analysis demonstrates that the incidence of delayed adverse effects in the SCT groups was comparable to that in the control groups across the included studies, [Risk Ratio (RR) = 0.97; 95% CI: 0.66, 1.40; *p* = 0.02] (Figure 10; Figure S12).

### 8.4. Subgroup Analysis

Based on the region the original study was conducted in, age, the patient count of the pooled studies, and other parameters, different subgroup analyses were performed to explore the observed heterogeneity for the primary outcomes of difference in the mean change in NIHSS score, mRS score, and BI (Supplementary Figures S13–S23).

## 9. Discussion

### 9.1. Primary Findings and Clinical Significance

Our updated meta-analysis of 18 RCTs provides compelling evidence supporting the safety and effectiveness of SCT in patients recovering from ischemic stroke. Although the NIHSS showed comparable results between the treatment and control groups, SCT demonstrated substantial improvements in terms of other functional and neurological outcomes, including BI, mRS, FMA scores, and infarct volume, suggesting potential neuroprotective effects. 

The safety profile analysis is very reassuring, with the most common adverse events being mild fever, headache, and fatigue, typically resolving without long-term consequences. Most studies have comparable or lower rates of adverse events in the treatment groups compared to controls. Although most of the trials, such as those conducted by Houkin et al. [22] and Hess et al. [32], highlighted the robust safety profile of SCT with serious adverse events rates of 52.8% and 34%, respectively, the higher rate of adverse reactions reported by Lee et al. [29] (75%) raises concerns regarding the underlying potential impact of different treatment procedures, patient populations, and reporting criteria. Although reported serious complications such as tumor formation, immune rejection, and venous thromboembolism remain rare, their seriousness and negative impact on patients may necessitate long-term follow-up [39].

### 9.2. Subgroup Analyses and Considerations for Implementation

The subgroup analysis results show crucial patterns that can be applied in future clinical practice. Trials with fewer patients yielded better outcomes, emphasizing the need for intensive monitoring. Additionally, more favorable results were noted with early treatment, particularly when treatment was administered within the first 2 months post-stroke. This suggests a probable benefit of early intervention through intravenous or intra-arterial routes with distinct risk profiles. However, the relationship between treatment timing and outcomes suggests that the therapeutic window can be more comprehensive than anticipated, but recovery outcomes are time-dependent. Recently, a study reported that subgroup analyses of the timing of MSCs administered within 2 weeks to 3 months post-stroke showed significant improvements in NIHSS scores [15].

### 9.3. Stroke Burden and Present Therapeutic Landscape

Stroke is still considered a leading cause of death and long-term disability around the globe, with an increasing burden noted in developing countries. In 2019, there were approximately 12.2 million new stroke cases, resulting in 143 million disability-adjusted life-years (DALYs) lost and 6.6 million deaths globally. Over the past 30 years, there has been a 70% increase in incident strokes, an 85% increase in prevalent strokes, and a 43% increase in stroke-related deaths [40,41]. Even though progress has been made in acute stroke management, mainly intravenous thrombolysis and mechanical thrombectomy, the major drawbacks are the narrow therapeutic time windows and minimal eligibility. Currently, guidelines proposed by the American Heart Association (AHA) indicate a time window for intravenous alteplase of 4.5 h, and for thrombectomy among selected patients of between 6 and 24 h [41,42]. However, a small portion of stroke patients can only receive the current standard of care, which highlights an unmet need for new treatment options. Conventional rehabilitation is only partially effective; 50% of all patients eventually regain some functional ability. All these drawbacks and the complex pathophysiology of brain damage due to stroke have shifted the search toward so-called innovative treatments, such as stem cell therapy [41,42,43].

### 9.4. Rationale and Mechanisms of Stem Cell Therapy

Recently, SCT has become an attractive treatment option; regarding the multi-modal mechanisms of stroke recovery explored since the early 2000s, studies began with autologous mesenchymal stem cells (MSCs) around 2005, which demonstrated their potential to repair damaged brain tissue and improve patient safety [13,43,44]. SCT for ischemic stroke involves a range of stem cell types such as MSC [13], Hematopoietic Stem Cells (HSCs) [31], and Endothelial Progenitor Cells (EPCs) [23], each offering unique mechanisms and therapeutic potential. 

The rationale behind using SCT for ischemic stroke is based on their ability to promote neuroprotection, neurogenesis, functional recovery, and immune system modulation [45]. Stem cells help to immunomodulate and reduce inflammation through the secretion of anti-inflammatory cytokines, such as interleukin-10 (IL-10) and transforming growth factor-beta (TGF-β), which help to develop a less hostile surrounding toward repairing damaged tissue caused by an inflammatory cascade following stroke. They influence the enhancement of endogenous neuroplasticity because they are likely to induce the formation of new neural connections [43,44].

Furthermore, stem cells enhance pre-existing vascular networks by secreting growth factors such as vascular endothelial growth factor (VEGF), which leads to the development of fresh blood vessels vasculature. These new avenues may support tissue regeneration by supplying increased blood circulation to areas affected by ischemia [32,33,40,41,42,43]. Neurons’ existence and activity are further promoted by the secreted neurotrophic factors. Moreover, though direct cell replacement may be minimal, MSCs can undergo differentiation into neural-like cells, which can support tissue repair. These dual mechanisms work together to promote recovery after a stroke, making MSCs especially attractive as a therapeutic intervention [43,44]. Furthermore, mesenchymal stem cells (MSCs) are widely studied due to their immunomodulatory properties and ability to secrete trophic factors that support neuronal survival and repair [45]. Neural stem cells (NSCs) have shown potential in differentiating into neurons and glial cells, contributing directly to tissue regeneration [46]. Although induced pluripotent stem cells (iPSCs) offer a patient-specific approach, reducing the risk of immune rejection, they require further safety evaluations due to the increased tumorigenesis risk [47]. While stem cells offer promising therapeutic benefits, they are not risk-free. Their diverse action mechanisms can also lead to serious adverse effects. While mesenchymal stem cells (MSCs) are generally associated with low immunogenicity and a favorable safety profile, some serious adverse effects have been reported, such as thromboembolism and fibrosis. Additionally, infusion-related reactions, such as fever, chills, and allergic responses, have been observed [48]. Neural stem cells (NSCs) may carry a higher risk of tumorigenesis in certain contexts, necessitating careful evaluation of their application [46]. While induced pluripotent stem cells (iPSCs) have demonstrated therapeutic potential in preclinical models, they require further clinical validation to assess long-term safety outcomes, as they were found to induce tumorigenesis, a strong immune response causing cell rejection [47]. 

MSC characterization criteria have been defined by the International Society for Cell & Gene Therapy (ISCT), providing a benchmark for standardizing clinical applications. The most recent position papers from stem cell committees, designed to spur increased rigor in the assessment and possible use of stem cell therapy, refer to the promise and attendant need for proper clinical evaluation [49,50]. The administration route plays a crucial role in maximizing the efficacy and safety of SCT. Among the most commonly used administrations are intravenous (IV) and intra-arterial (IA) delivery routes, each offering specific benefits and limitations. IV delivery is non-invasive and allows for the systemic distribution of stem cells, though a significant proportion of cells may become trapped in peripheral organs, reducing their targeting efficiency [15]. Additionally, IV administration has been associated with adverse events, such as thromboembolism, and infusion-related reactions, such as fever, chills, and allergic responses [48,51]. IA delivery, by contrast, facilitates the direct targeting of the ischemic brain region, which can enhance therapeutic outcomes. However, it requires greater technical skill and carries risks such as arterial damage or thrombosis [32]. For chronic stroke phases, intraparenchymal delivery is sometimes employed. Although this approach offers the most direct cell delivery, its invasive nature limits its use to specific cases, as noted by Steinberg et al. (2018) [52]. Emerging methods, such as intranasal delivery, have garnered attention for their ability to bypass the blood–brain barrier, enabling direct access to the brain. Preclinical studies, including those by Donega et al. (2013), suggest promising results; however, clinical data are sparse, necessitating further trials to assess safety and efficacy [53].

### 9.5. Patient-Reported Outcomes and Quality of Life

Beyond clinical and neurological outcomes, it is essential to assess the impact of stem cell therapy on patient-reported outcomes (PROs), including the quality of life and overall satisfaction. Evaluating PROs allows for a comprehensive understanding of how stem cell interventions influence daily functional abilities, psychological well-being, and social reintegration. SCT has shown promise in improving patient-reported outcomes, particularly by reducing disability and enhancing the quality of life, as assessed through the Barthel Index (BI) [4].

A meta-analysis evaluating the efficacy and safety of SCT in ischemic stroke demonstrated significant improvements in neurological function and the quality of life, as evidenced by increased BI scores [14]. Similarly, a systematic review reported that stem cell-based treatments contribute to the recovery of neurological deficits and daily living activities in ischemic stroke patients, reinforcing their positive impact on BI scores [54]. While current findings suggest that SCT enhances functional independence and the quality of life, evidence regarding its effect on survival rates remains inconclusive. Future research should incorporate standardized PRO measures to assess improvements in functional independence, fatigue levels, emotional well-being, and patient satisfaction with treatment outcomes.

### 9.6. Study Strengths in Comparison to the Recent Literature

Nagpal et al. (2017) [54] reported improvements in NIHSS, mRS, and BI, with a reassuring safety profile. However, the inclusion of various study designs and both stroke subtypes may have diluted the reliability of their conclusions. Therefore, our inclusion of pure RCTs with new outcome measures (FMA and infarct size) for ischemic stroke provides more compelling evidence of the efficacy and safety of SCT. Aligning with our findings, Ouyanget al.’s (2019) [55] assessment of the safety and efficacy of SCT in pre-clinical and clinical trials revealed a promising trend in the implemented stem cells in NIHSS, mRS, BI, and FMA. Although their search strategy was comprehensive, selective bias may have derived from the inclusion of English and Chinese studies only. In addition, the paucity of original data limited their ability to include some promising clinical trials, which is not the case in our study. 

Li et al. (2020) [14] demonstrated dynamic trends in the improvement in NHISS, mRS, and BI, with a favorable safety profile and functional independence. While their findings align with ours, their inclusion of non-randomized trials may limit their conclusion’s specificity. Our exploration of the sensitivity and heterogeneity analysis ensure the robustness of the assessments in a well-defined population. 

In contrast to our findings, Kumar et al. (2021) [56] reported no significant improvement in NIHSS, mRS, or BI following stem cell administration in ischemic stroke patients. This discrepancy may be partly due to the small number of RCTs included in their analysis. Our inclusion of larger, well-designed RCTs provides more robust conclusions. Xiong et al. (2024) [4] supported the efficacy and safety of SCT in ischemic stroke. However, their focus was limited to NHISS, mRS, and BI, whereas our study included FMA and infarct size as new outcome measures as well as a greater sample size, providing a more comprehensive review. 

## 10. Study Limitations

Several limitations should be considered when interpreting the results of this meta-analysis. Although we adopted a comprehensive approach, the heterogeneity of stem cell types, preparation protocols, characterization methods, and follow up periods across studies might have nurtured the influence on treatment outcomes. The negative confidence intervals reported in the infarct size suggest uncertainty in the direction of the effect, implying that SCT might lead to either a slight reduction or an increase in infarct volume, limiting the ability to draw a solid conclusion. Additionally, the long-term safety data beyond 24 months are scanty, compromising our ability to gain insight into the overall safety profile of stem cell therapy. Although our funnel plot analysis pointed towards slight publication bias, this should be taken into consideration due to the possible limitation of such an assumption. Wide disparities in the outcome measures across the studies further confounded any attempt at a meaningful comparison and pooling of results, undermining the precision of our estimates. This underscores another issue reflective of the limited data on cost-effectiveness regarding a sound appraisal of the economic implications of this therapy.

## 11. Future Directions and Research Priorities

Future research avenues in stem cell therapy for stroke should focus on several key areas required for the progression of the field toward the goals of optimal clinical outcomes. The most critical need would be the establishment of standard protocols for cell preparation and characterization to guarantee the same cellular products in all participants. Second, further optimization in this area includes studying various routes of administration and relative efficacies for the means and timing of delivery. Equally vital will be the determination of the patient subgroups most amenable to stem cell therapy and the various effects of different treatment dosages, so that treatments can be tailored specifically to maximize therapeutic outcomes. Lastly, longer-term follow-up assessments will offer information on stem cell therapy’s sustained benefits and possible late effects. If these research priorities are adequately addressed, they will help to establish stem cell therapy as a well-understood and reliably implemented treatment option for patients with stroke.

## 12. Conclusions

This meta-analysis provides compelling evidence that stem cell therapy is effective and safe for treating ischemic stroke patients. The significant effect sizes found in functional outcomes, especially those relating to motor function and activities of daily living, may indicate a potential place for stem cell therapy in treating stroke patients. The established efficacy and safety profiles encourage further development and appropriate additional research. Its clinical implementation, however, requires careful consideration regarding patient selection, time windows, and standardization of treatment protocols. The response patterns within different subgroups of patients might assist in refining the criteria for patient selection, whereas the areas of established uncertainty could guide future research efforts.

## Figures and Tables

**Table 3:** Safety outcomes.

Study (Author)	Route of Administration	Serious Adverse Effects, N (%)		Immediate Adverse Effects, N (%)		Delayed Adverse Effects, N (%)	
		SCT Group	Control Group	SCT Group	Control Group	SCT Group	Control Group
Bang et al. [13]	Intravenous	0%	N/A	0%	N/A	1 (20%) (Cellulitis = 1)	N/A
Bhasin et al. [25]	Intravenous	0%	0%	0%	0%	0%	0%
Lee et al. 2022 [35]	Intravenous	N/A	N/A	N/A	N/A	N/A	N/A
Jaillard et al. [34]	Intravenous	16 (Depression = 2, Urinary tract infection = 3, Humeral fracture = 1, Epileptic seizures = 6, Deep lower limb venous thrombosis = 1, Pneumonia = 2, Rotator cuff tear = 1),	23 (Recurrent Ischemic stroke = 2, TIA = 1, Urinary tract infection = 2, Crytpogenic fever = 1, Algodystrophia = 2,Humeral fracture = 2, Foot skin infection = 1, Epileptic seizures = 5, Pneumonia = 3, Gastrostomy = 1, Ankle sprain = 1, Atrial flutter = 1, Kidney pain = 1)	0%	0%	16 (Depression = 2, Urinary tract infection = 3, Humeral fracture = 1, Epileptic seizures = 6, Deep lower limb venous thrombosis = 1, Pneumonia = 2, Rotator cuff tear = 1)	23 (Recurrent ischemic stroke = 2, TIA = 1, Urinary tract infection = 2, Crytpogenic fever = 1, Algodystrophia = 2, Humeral fracture = 2, Foot skin infection = 1, Epileptic seizures = 5, Pneumonia = 3, Gastrostomy = 1, Ankle sprain = 1, Atrial flutter = 1, Kidney pain = 1)
Bhatia et al. [28]	Intra-arterial	2 (20%) (Death = 1, New Infarct = 1)	2 (20%) (Death = 2)	0%	0%	0%	0%
Chen et al. [30]	Subcutaneously	0%	0%	0%	0%	0%	0%
Fang et al. [23]	Intravenous	0%	0%	0%	0%	0%	0%
Wang et al. [26]	Olfactory sub-mucosa Injection	0%	0%	0%	0%	0%	0%
Prasad et al. [31]	Intravenous	12 (20%) (Pneumonitis = 1, Fracture in lower limb = 2, Death = 8, Bilateral lower limb ischemia = 1)	8 (13.3%) (Hypertension = 1, Fracture in lower limb = 1, Septicaemia with shock = 1, Death = 5)	0%	0%	61 (33%) (Rise in urea > 2.77 mmol/l = 2, Hematological = 10, Hepatic = 22, Serious deterioration in sensorium = 1, Pneumonitis = 1, Fever = 1, Hyperglycaemia = 1, Bilateral lower limb ischemia = 1, Frozen shoulder = 2, Traumatic injury = 1, Fracture in lower limb = 2, Death = 8, CNS = 6, GI = 3)	60 (36%) (Rise in urea > 2.77 mmol/l = 1, Hematological = 20, Hepatic = 13, Hypotension = 1, Edoema = 1, Hyperglycaemia = 3, Hypertension = 1, Septicaemia with shock = 1, Traumatic injury = 1, Fracture in lower limb = 1, Death = 5, CNS = 7, GI = 4, Increase in standardized uptake value of breast = 1, Uterine lesion on PET scan = 1)
Lee et al. 2010 [29]	Intravenous	12 (75%) (Death = 4, Small mass at lateral malleolus of the left ankle = 1, Seizure = 3, Recurrent vascular episode = 4)	29 (80.5%) (Death = 21, Seizure = 5, Recurrent vascular episode = 3)	8 (50%) (Recurrent stroke = 2, Myocardial infarction or angina = 1, Peripheral artery occlusive disease = 1, Infection = 3, Liver enzyme elevation = 1)	15 (36.1%) (Recurrent stroke = 1, Myocardial infarction or angina = 2, Infection = 9, Acute renal failure = 1, Liver enzyme elevation = 2)	10 (62.5%) (Benign mass = 1, Seizure = 3, Neuropyschological illness = 6)	14 (38.9%) (Systemic cancer = 1, Benign mass = 1, Seizure = 5, Neuropyschological illness = 7)
Savitz et al. [33]	Intravenous	16 (51.76%) (Convulsion = 2, Cerebral hemorrhage = 1, Deep vein thrombosis = 2, Hypertension = 2, Hypotension = 1, Angina = 1, Sick sinus syndrome = 1, Pulmonary embolism = 2, Urinary tract infection = 1, Chest pain = 1, Anxiety = 1, Craniectomy = 1)	10 (52.6%) (Cerebrovascular accident = 1, Hemorrhagic transformation = 1, Syncope = 1, Tachycardia = 1, Ventricular tachycardia = 1, Dyspnea = 1, Pneumonia = 1, Thrombocytopenia = 1, Retinal artery embolism = 1, Astrocytoma = 1)	0%	0%	N/A	N/A
Chung et al. [27]	Intravenous	0%	0%	0%	0%	0%	0%
Hess et al. [32]	Intravenous	34%	39%	99%	97%	33 (21.5%) (Life-threatening adverse events or death = 8, Infections = 25)	44 (72.1%) (Life-threatening adverse events or death = 15, Infections = 29)
Celis-Ruiz et al. [21]	intravenous	0%	4 (44.4%) (Deaths = 1, other = 3)	0%	0%	0%	1 (10%) (Death = 1)
Moniche et al. [36]	Intra-arterial	0%	0%	0%	0%	0%	0%
Law et al. [24]	Intravenous	0%	0%	0%	0%	0%	0%
Laskowitz et al. [37]	Intravenous	1 (2.1%) (1 Thromboembolic Event)	0.00%	0%	0%	0%	4 (15.4%) (Hypertension = 3, Seizure = 1)
Houkin et al. [22]	Intravenous	55 (52.8%) (Death = 7, infections = 48)	45 (44.1%) (Death = 6, infections = 38, SAE occurring within 7 days after treatment related to the investigational product = 1)	N/A	N/A	55 (52.8%) (Death = 7, Infections = 48)	45 (44.1%) (Death = 6, Infections = 38, SAEs occurring within 7 days after treatment related to the investigational product = 1)

SCT: Stem cell therapy; N/A: Not applicable; TIA; Transient ischemic attack; CNS Central nervous system; GI: Gastrointestinal; SAE: Serious adverse effects.

## Data Availability

All data generated or analyzed during this study are included in this published article [and its Supplementary Information Files].

## Supplementary Materials

The following supporting information can be downloaded at: https://www.mdpi.com/article/10.3390/jcm14062118/s1, Figure S1: Funnel plot for the difference between the mean change in the NIHSS score; Figure S2. Funnel plot for the difference between the mean change in the mRS score; Figure S3 Funnel plot for the difference between the mean change in the BI.; Figure S4. Funnel plot for the difference between the mean change in the FMA score; Figure S5. Funnel plot for the difference between the mean change in the infarct volume; Figure S6. Funnel plot for the mean difference of the safety outcomes; Figure S7. Leave one out analysis for the difference between the mean change in the NIHSS score; Figure S8. Leave one out analysis for the difference between the mean change in the mRS score; Figure S9. Leave one out analysis for the difference between the mean change in the BI; Figure S10. Forest plot of the incidence of serious adverse events in (A) SCT group and (B) placebo group and (C) difference between SCT and control group; Figure S11. Forest plot of the incidence of immediate adverse events in (A) SCT and (B) placebo groups and (C) The difference between SCT and placebo groups; Figure S12. Forest plot of the incidence of delayed adverse events in (A) SCT and (B) placebo groups and (C) Difference between SCT and placebo group; Figure S13. Sub-grouping of the difference between the mean change in the NIHSS score by the countries of the original papers; Figure S14. Sub-grouping of the difference between the mean change in the NIHSS score by the age distribution of the studied population; Figure S15. Sub-grouping of the difference between the mean change in the NIHSS score by the patient count; Figure S16. Sub-grouping of the difference between the mean change in the NIHSS score by the gender distribution in the studies; Figure S17. Sub-grouping of the difference between the mean change in the mRS score by the duration of stroke in the original papers; Figure S18. Sub-grouping of the difference between the mean change in the mRS score by the patient count of the original papers; Figure S19. Sub-grouping of the difference between the mean change in the mRS score by the patient’s gender distribution of the original papers; Figure S20. Sub-grouping of the difference between the mean change in the Barthel index by the patient’s country distribution of the original papers; Figure S21. Sub-grouping of the difference between the mean change in the Barthel index by the patient’s gender distribution of the original papers; Figure S22. Sub-grouping of the difference between the mean change in the Barthel index by the duration of stroke in the original papers; Figure S23. Sub-grouping of the difference between the mean change in the Barthel index by the duration of follow-up in the original papers.

## References

[1] Barthels D., Das H. (2020). Current advances in ischemic stroke research and therapies. Biochim. Biophys. Acta (BBA) Mol. Basis Dis..

[2] Feske S.K. (2021). Ischemic Stroke. Am. J. Med..

[3] GBD 2019 Stroke Collaborators (2021). Global, regional, and national burden of stroke and its risk factors, 1990–2019: A systematic analysis for the Global Burden of Disease Study 2019. Lancet Neurol..

[4] Xiong Y., Guo X., Gao W., Ke C., Huang X., Pan Z., Chen C., Zheng H., Hu W., Zheng F. (2024). Efficacy and safety of stem cells in the treatment of ischemic stroke: A meta-analysis. Medicine.

[5] Chavez L.M., Huang S.S., MacDonald I., Lin J.G., Lee Y.C., Chen Y.H. (2017). Mechanisms of Acupuncture Therapy in Ischemic Stroke Rehabilitation: A Literature Review of Basic Studies. Int. J. Mol. Sci..

[6] Hankey G.J. (2003). Long-Term Outcome after Ischaemic Stroke/Transient Ischaemic Attack. Cerebrovasc. Dis..

[7] Gómez-Outes A., Alcubilla P., Calvo-Rojas G., Terleira-Fernández A.I., Suárez-Gea M.L., Lecumberri R., Vargas-Castrillón E. (2021). Meta-Analysis of Reversal Agents for Severe Bleeding Associated with Direct Oral Anticoagulants. J. Am. Coll. Cardiol..

[8] Hovhannisyan L., Khachatryan S., Khamperyan A., Matinyan S. (2024). A review and meta-analysis of stem cell therapies in stroke patients: Effectiveness and safety evaluation. Neurol. Sci..

[9] Kalladka D., Sinden J., Pollock K., Haig C., McLean J., Smith W., McConnachie A., Santosh C., Bath P.M., Dunn L. (2016). Human neural stem cells in patients with chronic ischaemic stroke (PISCES): A phase 1, first-in-man study. Lancet.

[10] Takahashi K., Yamanaka S. (2013). Induced pluripotent stem cells in medicine and biology. Development.

[11] Gautam J., Alaref A., Hassan A., Kandel R.S., Mishra R., Jahan N. (2020). Safety and Efficacy of Stem Cell Therapy in Patients with Ischemic Stroke. Cureus.

[12] Zheng H., Zhang B., Chhatbar P.Y., Dong Y., Alawieh A., Lowe F., Hu X., Feng W. (2018). Mesenchymal Stem Cell Therapy in Stroke: A Systematic Review of Literature in Pre-Clinical and Clinical Research. Cell Transplant..

[13] Bang O.Y., Lee J.S., Lee P.H., Lee G. (2005). Autologous mesenchymal stem cell transplantation in stroke patients. Ann. Neurol..

[14] Li Z., Dong X., Tian M., Liu C., Wang K., Li L., Liu Z., Liu J. (2020). Stem cell-based therapies for ischemic stroke: A systematic review and meta-analysis of clinical trials. Stem Cell Res. Ther..

[15] Shen Z., Tang X., Zhang Y., Jia Y., Guo X., Guo X., Bao J., Xie X., Xing Y., Xing J. (2024). Efficacy and safety of mesenchymal stem cell therapies for ischemic stroke: A systematic review and meta-analysis. Stem Cells Transl. Med..

[16] PRISMA Group (2020). PRISMA Statement [Internet]. https://www.prisma-statement.org/.

[17] Ouzzani M., Hammady H., Fedorowicz Z., Elmagarmid A. (2016). Rayyan—A web and mobile app for systematic reviews. Syst. Rev..

[18] RevMan Group (2020). Cochrane. RevMan5.4_Statment. https://training.cochrane.org/online-learning/core-software.

[19] Higgins J.P.T., Thompson S.G., Deeks J.J., Altman D.G. (2003). Measuring inconsistency in meta-analyses. BMJ.

[20] Cumpston M., Li T., Page M., Chandler J., Welch V., Higgins J.P., Thomas J. (2019). Updated guidance for trusted systematic reviews: A new edition of the Cochrane Handbook for Systematic Reviews of Interventions. Cochrane Database Syst. Rev..

[21] de Celis-Ruiz E., Fuentes B., Alonso de Leciñana M., Gutiérrez-Fernández M., Borobia A.M., Gutiérrez-Zúñiga R., Ruiz-Ares G., Otero-Ortega L., Laso-García F., Gómez-de Frutos M.C. (2022). Final Results of Allogeneic Adipose Tissue-Derived Mesenchymal Stem Cells in Acute Ischemic Stroke (AMASCIS): A Phase II, Randomized, Double-Blind, Placebo-Controlled, Single-Center, Pilot Clinical Trial. Cell Transplant..

[22] Houkin K., Houkin K., Osanai T., Osanai T., Uchiyama S., Uchiyama S., Minematsu K., Minematsu K., Taguchi A., Taguchi A. (2024). Allogeneic Stem Cell Therapy for Acute Ischemic Stroke: The Phase 2/3 TREASURE Randomized Clinical Trial. JAMA Neurol..

[23] Fang J., Guo Y., Tan S., Li Z., Xie H., Chen P., Wang K., He Z., He P., Ke Y. (2019). Autologous Endothelial Progenitor Cells Transplantation for Acute Ischemic Stroke: A 4-Year Follow-Up Study. Stem Cells Transl. Med..

[24] Law Z.K., Tan H.J., Chin S.P., Wong C.Y., Yahya W.N.N.W., Muda A.S., Zakaria R., Ariff M.I., Ismail N.A., Cheong S.K. (2021). The effects of intravenous infusion of autologous mesenchymal stromal cells in patients with subacute middle cerebral artery infarct: A phase 2 randomized controlled trial on safety, tolerability and efficacy. Cytotherapy.

[25] Bhasin A., Srivastava M.P., Mohanty S., Vivekanandhan S., Sharma S., Kumaran S., Bhatia R. (2016). Paracrine Mechanisms of Intravenous Bone Marrow-Derived Mononuclear Stem Cells in Chronic Ischemic Stroke. Cerebrovasc. Dis. Extra.

[26] Wang Y., Guo X., Liu J., Zheng Z., Liu Y., Gao W., Xiao J., Liu Y., Li Y., Tang M. (2020). Olfactory ensheathing cells in chronic ischemic stroke: A phase 2, double-blind, randomized, controlled trial. J. Neurorestoratol..

[27] Chung J.-W., Chang W.H., Bang O.Y., Moon G.J., Kim S.J., Kim S.-K., Lee J.S., Sohn S.-I., Kim Y.-H. (2021). Efficacy and Safety of Intravenous Mesenchymal Stem Cells for Ischemic Stroke. Neurology.

[28] Bhatia V., Gupta V., Khurana D., Sharma R., Khandelwal N. (2018). Randomized Assessment of the Safety and Efficacy of Intra-Arterial Infusion of Autologous Stem Cells in Subacute Ischemic Stroke. AJNR Am. J. Neuroradiol..

[29] Lee J.S., Hong J.M., Moon G.J., Lee P.H., Ahn Y.H., Bang O.Y. (2010). A long-term follow-up study of intravenous autologous mesenchymal stem cell transplantation in patients with ischemic stroke. Stem Cells.

[30] Chen D.C., Lin S.Z., Fan J.R., Lin C.H., Lee W., Lin C.C., Liu Y.J., Tsai C.H., Chen J.C., Cho D.Y. (2014). Intracerebral implantation of autologous peripheral blood stem cells in stroke patients: A randomized phase II study. Cell Transplant..

[31] Prasad K., Sharma A., Garg A., Mohanty S., Bhatnagar S., Johri S., Singh K.K., Nair V., Sarkar R.S., Gorthi S.P. (2014). Intravenous autologous bone marrow mononuclear stem cell therapy for ischemic stroke: A multicentric, randomized trial. Stroke.

[32] Hess D.C., Wechsler L.R., Clark W.M., Savitz S.I., Ford G.A., Chiu D., Yavagal D.R., Uchino K., Liebeskind D.S., Auchus A.P. (2017). Safety and efficacy of multipotent adult progenitor cells in acute ischaemic stroke (MASTERS): A randomised, double-blind, placebo-controlled, phase 2 trial. Lancet Neurol..

[33] Savitz S.I., Yavagal D., Rappard G., Likosky W., Rutledge N., Graffagnino C., Alderazi Y., Elder J.A., Chen P.R., Budzik R.F. (2019). A Phase 2 Randomized, Sham-Controlled Trial of Internal Carotid Artery Infusion of Autologous Bone Marrow-Derived ALD-401 Cells in Patients with Recent Stable Ischemic Stroke (RECOVER-Stroke). Circulation.

[34] Jaillard A., Hommel M., Moisan A., Zeffiro T.A., Favre-Wiki I.M., Barbieux-Guillot M., Vadot W., Marcel S., Lamalle L., Grand S. (2020). Autologous Mesenchymal Stem Cells Improve Motor Recovery in Subacute Ischemic Stroke: A Randomized Clinical Trial. Transl. Stroke Res..

[35] Lee J., Chang W.H., Chung J.W., Kim S.J., Kim S.K., Lee J.S., Sohn S.I., Kim Y.H., Bang O.Y. (2022). Efficacy of Intravenous Mesenchymal Stem Cells for Motor Recovery After Ischemic Stroke: A Neuroimaging Study. Stroke.

[36] Moniche F., Cabezas-Rodriguez J.A., Valverde R., Escudero-Martinez I., Lebrato-Hernandez L., Pardo-Galiana B., Ainz L., Medina-Rodriguez M., de la Torre J., Escamilla-Gomez V. (2023). Safety and efficacy of intra-arterial bone marrow mononuclear cell transplantation in patients with acute ischaemic stroke in Spain (IBIS trial): A phase 2, randomised, open-label, standard-of-care controlled, multicentre trial. Lancet Neurol..

[37] Laskowitz D.T., Troy J., Poehlein E., Bennett E.R., Shpall E.J., Wingard J.R., Freed B., Belagaje S.R., Khanna A., Jones W. (2024). A Randomized, Placebo-Controlled, Phase II Trial of Intravenous Allogeneic Non-HLA Matched, Unrelated Donor, Cord Blood Infusion for Ischemic Stroke. Stem Cells Transl. Med..

[38] Sterne J.A.C., Savović J., Page M.J., Elbers R.G., Blencowe N.S., Boutron I., Cates C.J., Cheng H.Y., Corbett M.S., Eldridge S.M. (2019). RoB 2: A revised tool for assessing risk of bias in randomised trials. BMJ.

[39] Trounson A., Thakar R.G., Lomax G., Gibbons D. (2011). Clinical trials for stem cell therapies. BMC Med..

[40] eClinicalMedicine (2023). The rising global burden of stroke. eClinicalMedicine.

[41] Pu L., Wang L., Zhang R., Zhao T., Jiang Y., Han L. (2023). Projected Global Trends in Ischemic Stroke Incidence, Deaths and Disability-Adjusted Life Years From 2020 to 2030. Stroke.

[42] Marín-Medina D.S., Arenas-Vargas P.A., Arias-Botero J.C., Gómez-Vásquez M., Jaramillo-López M.F., Gaspar-Toro J.M. (2024). New approaches to recovery after stroke. Neurol. Sci..

[43] AAderinto N., Olatunji G., Kokori E., Babalola A.E., Yusuf I.A., Apampa O.O., Ukoaka B.M., Aboje J.E., Adefusi T., Moradeyo A. (2024). Stem cell therapies in stroke rehabilitation: A narrative review of current strategies and future prospects. Egypt J. Neurol. Psychiatr. Neurosurg..

[44] Zhang Y., Dong N., Hong H., Qi J., Zhang S., Wang J. (2022). Mesenchymal Stem Cells: Therapeutic Mechanisms for Stroke. Int. J. Mol. Sci..

[45] Li W., Shi L., Hu B., Hong Y., Zhang H., Li X., Zhang Y. (2021). Mesenchymal Stem Cell-Based Therapy for Stroke: Current Understanding and Challenges. Front. Cell. Neurosci..

[46] Kaneko N., Kako E., Sawamoto K. (2011). Prospects and Limitations of Using Endogenous Neural Stem Cells for Brain Regeneration. Genes.

[47] Scheiner Z.S., Talib S., Feigal E.G. (2013). The Potential for Immunogenicity of Autologous Induced Pluripotent Stem Cell-derived Therapies. J. Biol. Chem..

[48] Petrou P., Gothelf Y., Argov Z., Gotkine M., Levy Y.S., Kassis I., Vaknin-Dembinsky A., Ben-Hur T., Offen D., Abramsky O. (2016). Safety and Clinical Effects of Mesenchymal Stem Cells Secreting Neurotrophic Factor Transplantation in Patients with Amyotrophic Lateral Sclerosis: Results of Phase 1/2 and 2a Clinical Trials. JAMA Neurol..

[49] Ikonomou L., Cuende N., Forte M., Grilley B.J., Levine A.D., Munsie M., Rasko J.E., Turner L., Bidkhori H.R., Ciccocioppo R. (2023). International Society for Cell & Gene Therapy Position Paper: Key considerations to support evidence-based cell and gene therapies and oppose marketing of unproven products. Cytotherapy.

[50] Renesme L., Pierro M., Cobey K.D., Mital R., Nangle K., Shorr R., Lalu M.M., Thébaud B. (2022). Definition and Characteristics of Mesenchymal Stromal Cells in Preclinical and Clinical Studies: A Scoping Review. Stem Cells Transl. Med..

[51] Kawabori M., Shichinohe H., Kuroda S., Houkin K. (2020). Clinical Trials of Stem Cell Therapy for Cerebral Ischemic Stroke. Int. J. Mol. Sci..

[52] Steinberg G.K., Kondziolka D., Wechsler L.R., Lunsford L.D., Coburn M.L., Billigen J.B., Kim A.S., Johnson J.N., Bates D., King B. (2016). Clinical Outcomes of Transplanted Modified Bone Marrow-Derived Mesenchymal Stem Cells in Stroke: A Phase 1/2a Study. Stroke.

[53] Donega V., van Velthoven C.T.J., Nijboer C.H., van Bel F., Kas M.J.H., Kavelaars A., Heijnen C.J. (2013). Intranasal mesenchymal stem cell treatment for neonatal brain damage: Long-term cognitive and sensorimotor improvement. PLoS ONE.

[54] Nagpal A., Choy F.C., Howell S., Hillier S., Chan F., Hamilton-Bruce M.A., Koblar S.A. (2017). Safety and effectiveness of stem cell therapies in early-phase clinical trials in stroke: A systematic review and meta-analysis. Stem Cell Res. Ther..

[55] Ouyang Q., Li F., Xie Y., Han J., Zhang Z., Feng Z., Su D., Zou X., Cai Y., Zou Y. (2019). Meta-Analysis of the Safety and Efficacy of Stem Cell Therapies for Ischemic Stroke in Preclinical and Clinical Studies. Stem Cells Dev..

[56] Kumar A., Rawat D., Prasad K. (2021). Stem Cell Therapy in Ischemic Stroke: A Systematic Review and Meta-Analysis of Randomized Controlled Trials. Ann. Indian Acad. Neurol..

