# Supplementary material for: Safety and Efficacy of Stem Cell Therapy in Ischemic Stroke: A Comprehensive Systematic Review and Meta-Analysis
Source: J Clin Med. 2025 Mar 20;14(6):2118. doi: 10.3390/jcm14062118 (PMC11943215; doi:10.3390/jcm14062118)
Supplement: Supplementary file 1 [file jcm-14-02118-s001.zip › jcm-3531848-supplementary.pdf]

Supplemental Online Content

Systematic Review

Safety and Efficacy of Stem Cell Therapy in Ischemic Stroke: A Comprehensive Systematic Review and Meta-Analysis

Abdulrahim Saleh Alrasheed [1,*], Tala Abdullah Aljahdali [2], Israa Aqeel Alghafli [3], Ghadeer Aqeel Alghafli [4], Majd Fouad Almuslim [4], Noor Mohammad AlMohish [5] and Majed Mohammad Alabdali [6]

1 Department of Neurosurgery, College of Medicine, King Faisal University, AlAhsa 31982, Saudi Arabia
2 College of Medicine, King Saud bin Abdulaziz University for Health Sciences, Riyadh 11426, Saudi Arabia; aljahdali185@ksau-hs.edu.sa
3 College of Medicine, King Faisal University, AlAhsa 31982, Saudi Arabia; 221427343@student.kfu.edu.sa
4 College of Applied Medical Sciences, Nursing Department, King Faisal University, AlAhsa 31982, Saudi Arabia; 220026718@student.kfu.edu.sa (G.A.A.); 220011200@student.kfu.edu.sa (M.F.A.)
5 Neurology Department, King Fahad Hospital of the University, Imam Abdulrahman Bin Faisal University, Khobar 31441, Saudi Arabia; nmalmohish@iau.edu.sa
6 Neurology Department, College of Medicine, Imam Abdulrahman Bin Faisal University, Khobar 31441, Saudi Arabia; mmalabdali@iau.edu.sa

***** Correspondence: 221414880@student.kfu.edu.sa or abdulrhim2003@hotmail.com

Figure S1. Funnel plot for the difference between the mean change in the NIHSS score.

Figure S2. Funnel plot for the difference between the mean change in the mRS score.

Figure S3 Funnel plot for the difference between the mean change in the BI.

Figure S4. Funnel plot for the difference between the mean change in the FMA score.

Figure S5. Funnel plot for the difference between the mean change in the infarct volume.

Figure S6. Funnel plot for the mean difference of the safety outcomes.

Figure S7. Leave one out analysis for the difference between the mean change in the NIHSS score.

Figure S8. Leave one out analysis for the difference between the mean change in the mRS score.

Figure S9. Leave one out analysis for the difference between the mean change in the BI.

Figure S10. Forest plot of the incidence of serious adverse events in A) SCT group and B) placebo group and C) difference between SCT and control group.

Figure S11. Forest plot of the incidence of immediate adverse events in A) SCT and B) placebo groups and C) The difference between SCT and placebo groups.

Figure S12. Forest plot of the incidence of delayed adverse events in a) SCT and B) placebo groups and C) Difference between SCT and placebo group.

Figure S13. Sub-grouping of the difference between the mean change in the NIHSS score by the countries of the original papers.

Figure S14. Sub-grouping of the difference between the mean change in the NIHSS score by the age distribution of the studied population.

Figure S15. Sub-grouping of the difference between the mean change in the NIHSS score by the patient count.

Figure S16. Sub-grouping of the difference between the mean change in the NIHSS score by the gender distribution in the studies.

Figure S17. Sub-grouping of the difference between the mean change in the mRS score by the duration of stroke in the original papers.

Figure S18. Sub-grouping of the difference between the mean change in the mRS score by the patient count of the original papers.

Figure S19. Sub-grouping of the difference between the mean change in the mRS score by the patient's gender distribution of the original papers.

Figure S20. Sub-grouping of the difference between the mean change in the Barthel index by the patient's country distribution of the original papers.

Figure S21. Sub-grouping of the difference between the mean change in the Barthel index by the patient's gender distribution of the original papers.

Figure S22. Sub-grouping of the difference between the mean change in the Barthel index by the duration of stroke in the original papers.

Figure S23. Sub-grouping of the difference between the mean change in the Barthel index by the duration of follow-up in the original papers.

Publication bias

The funnel plots for the primary outcomes, including the comparison of mean change in the National Institutes of Health Stroke Scale (NIHSS), the modified Rankin Scale (mRS), Barthel Index (BI), Fugl-Meyer Assessment (FMA), infarct volume, and safety outcomes of the pooled studies, were symmetric and suggested no obvious publication bias in reporting the outcomes in the patients by the included studies. The funnel plots are given in (Figures S 1-6).

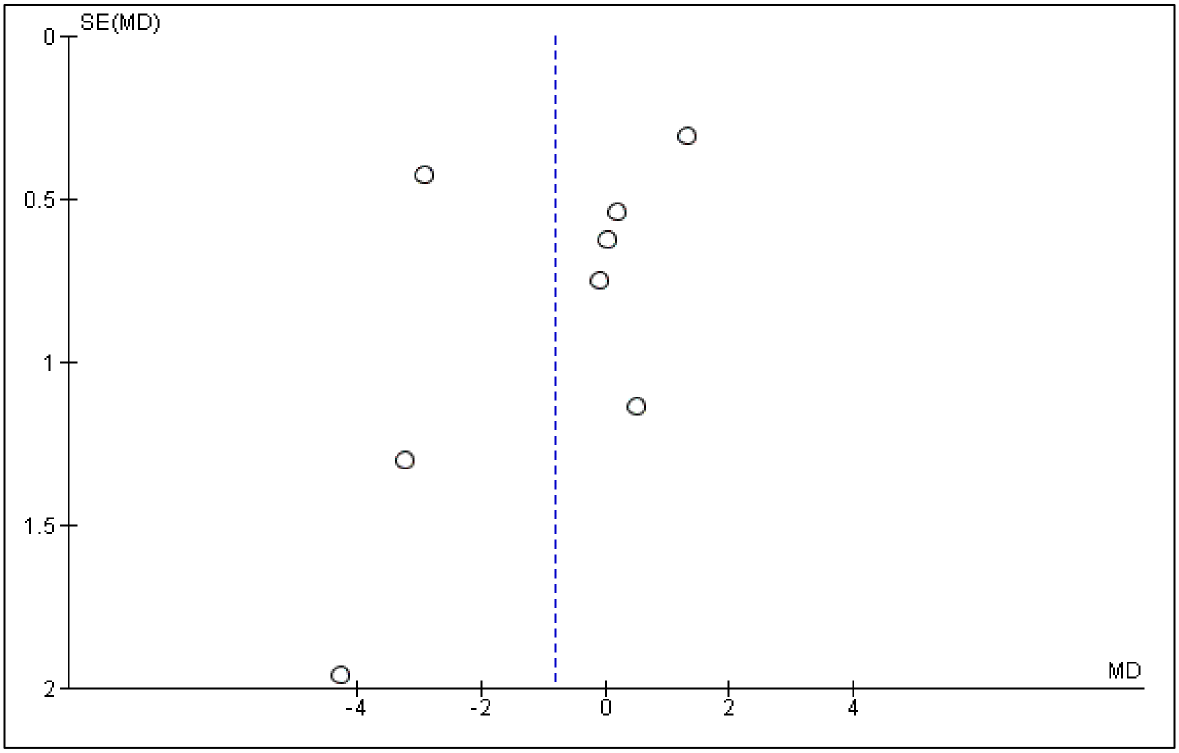

Figure S1: Funnel plot for the difference between the mean change in the NIHSS score.

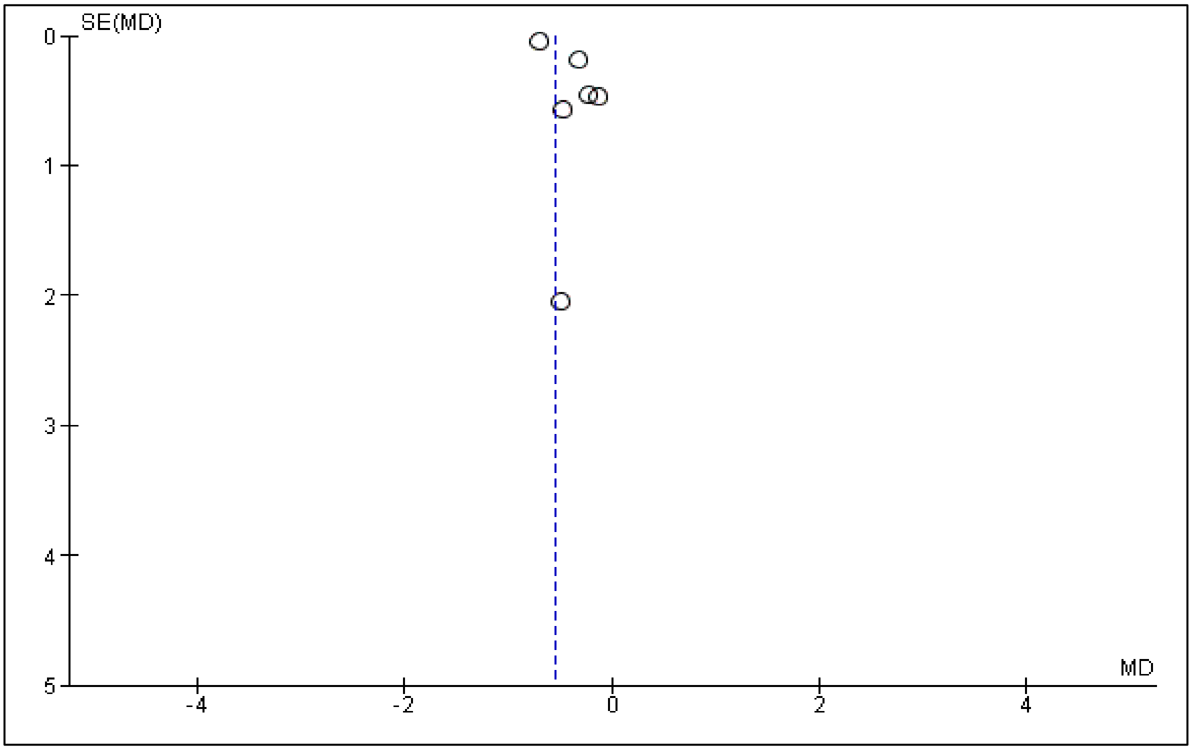

Figure S2: Funnel plot for the difference between the mean change in the mRS score.

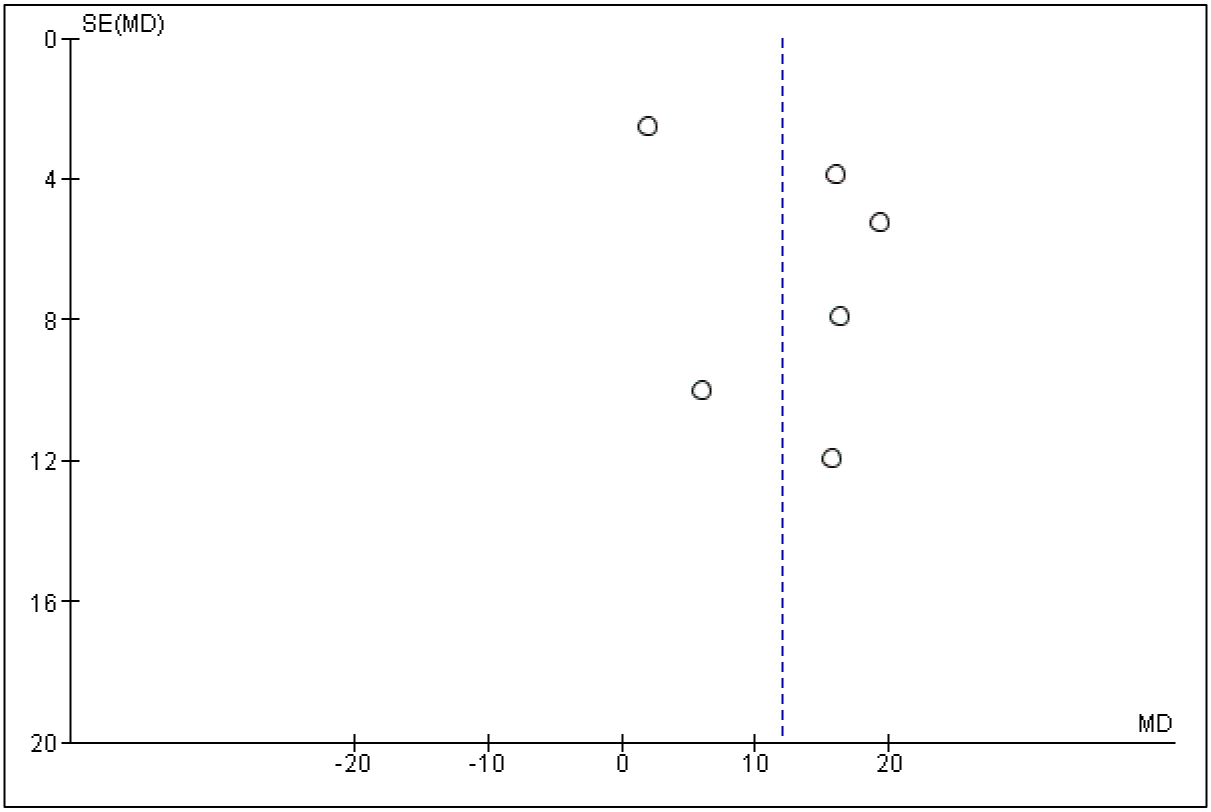

Figure S3: Funnel plot for the difference between the mean change in the BI.

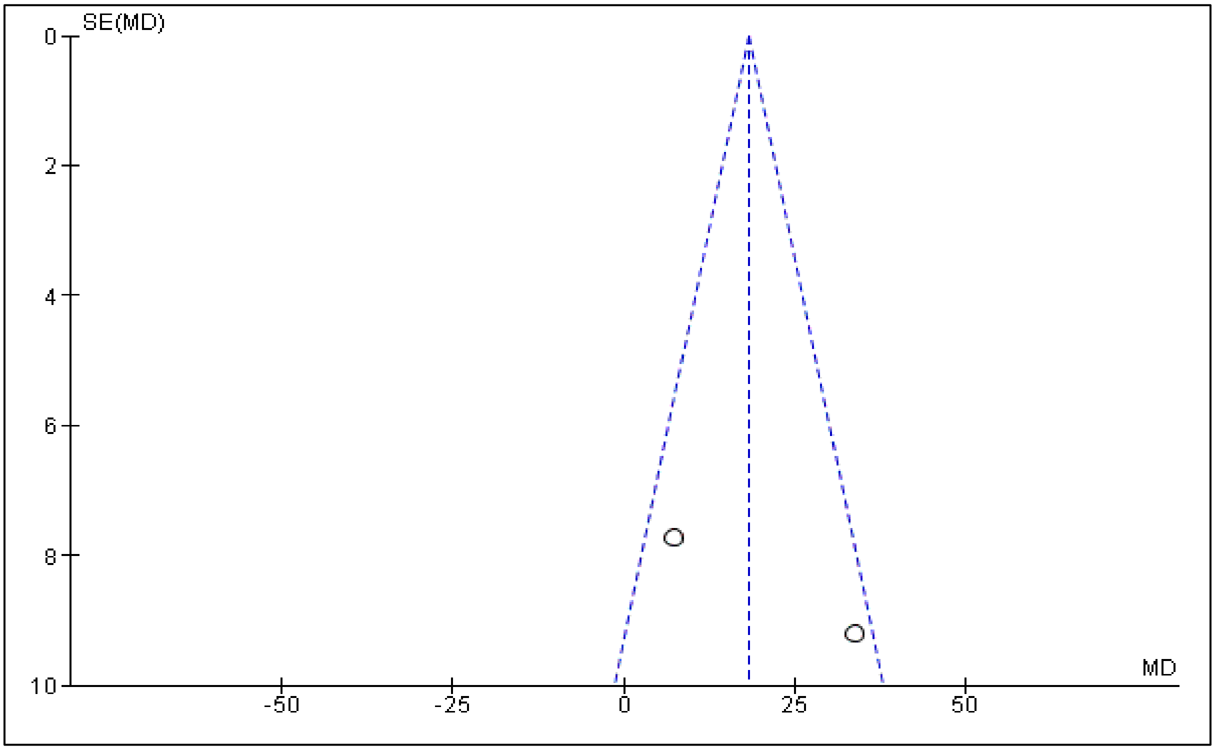

Figure S4: Funnel plot for the difference between the mean change in the FMA score.

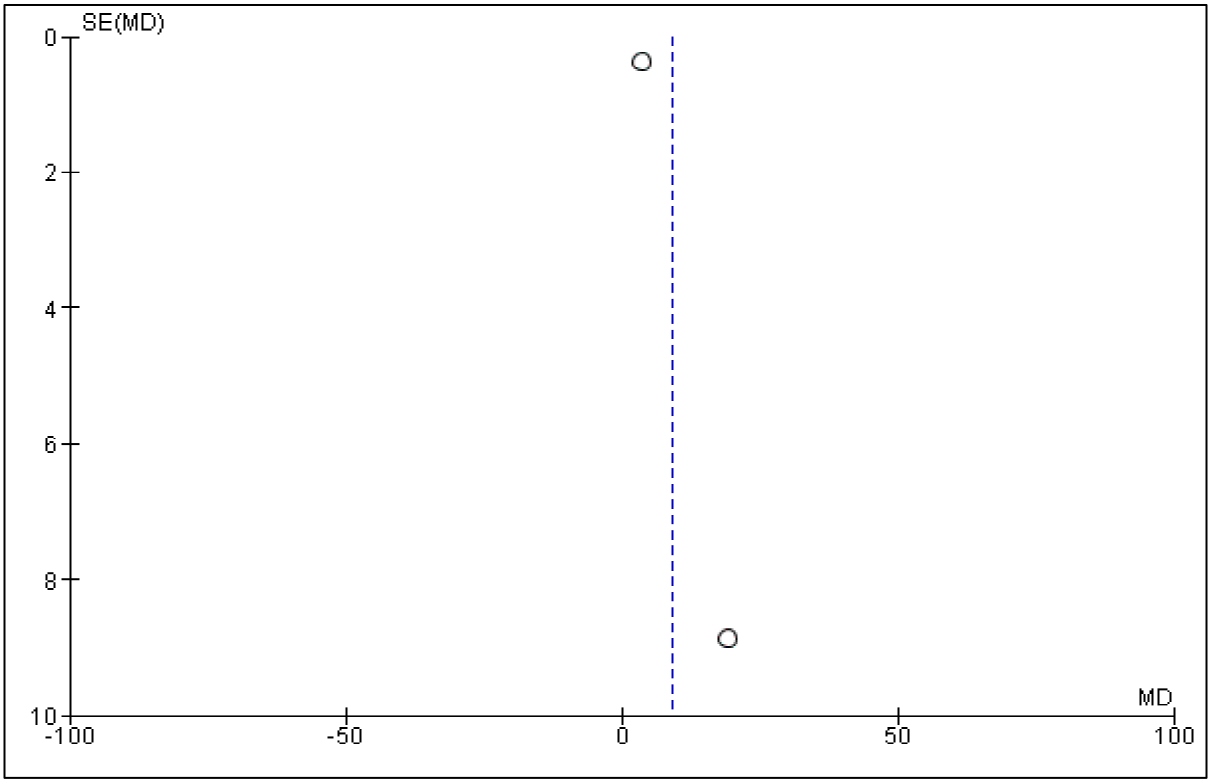

Figure S5: Funnel plot for the difference between the mean change in the infarct volume.

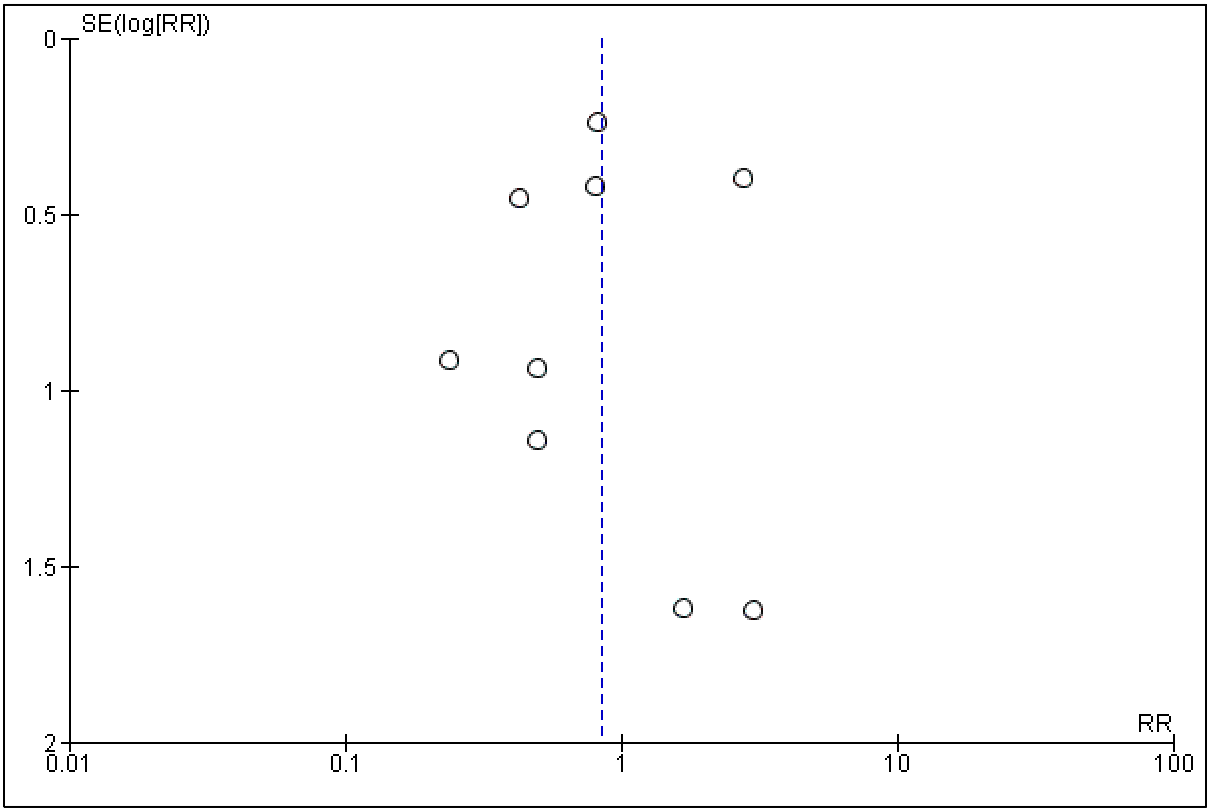

Figure S6: Funnel plot for the mean difference of the safety outcomes.

Leave one out analysis

Leave-one-out analysis was performed for all the primary outcomes and could not demonstrate any heterogeneity in the findings of the pooled analysis of the included studies. The plots and summaries of the leave-one-out analysis are given in (Figure S7-9) for the difference in the mean change of NIHSS score, mRS score, and BI.

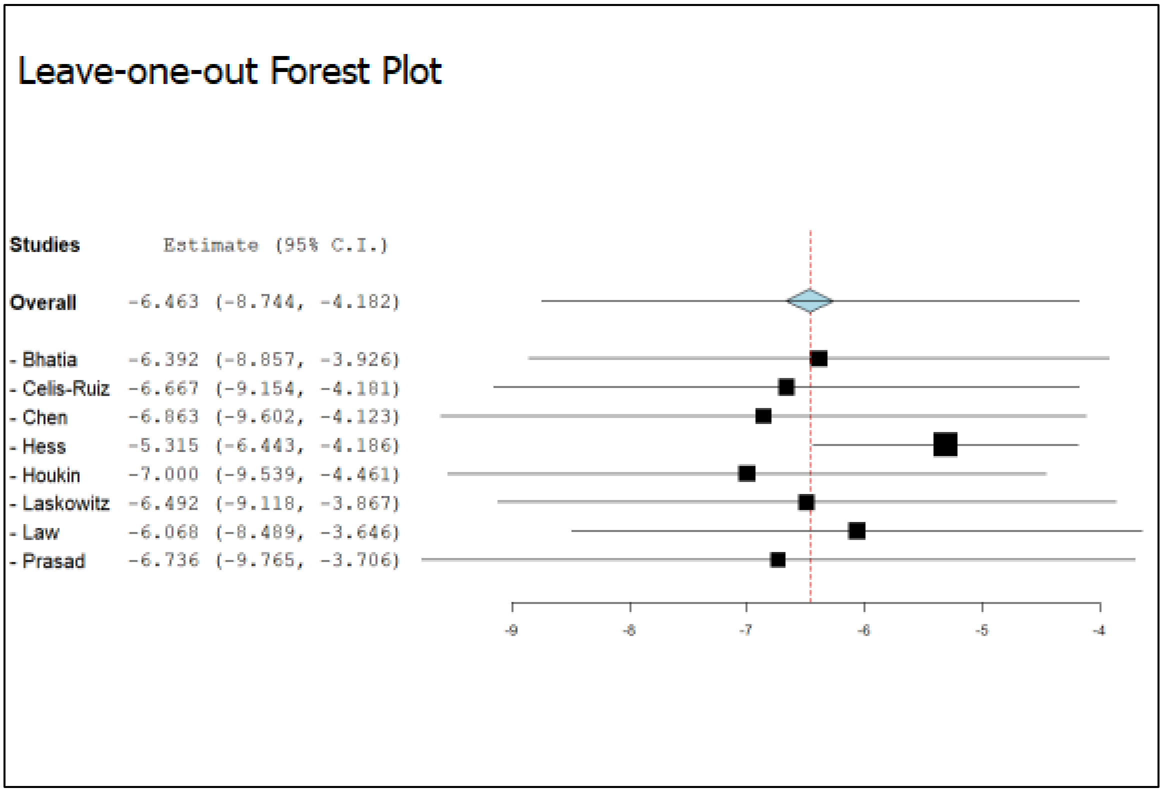
Leave-one-out Forest Plot
Studies
Estimate (95% C.I.)
Overall -6.463 (-8.744, -4.182)
- Bhatia -6.392 (-8.857, -3.926)
- Celis-Ruiz -6.667 (-9.154, -4.181)
- Chen -6.863 (-9.602, -4.123)
- Hess -5.315 (-6.443, -4.186)
- Houkin -7.000 (-9.539, -4.461)
- Laskowitz -6.492 (-9.118, -3.867)
- Law -6.068 (-8.489, -3.646)
- Prasad -6.736 (-9.765, -3.706)
-9
-8
-7
-6
-5
-4

- Bhatia	-6.392	-8.857	-3.926	1.258	< 0.001
- Celis-Ruiz	-6.667	-9.154	-4.181	1.269	< 0.001
- Chen	-6.863	-9.602	-4.123	1.398	< 0.001
- Hess	-5.315	-6.443	-4.186	0.576	< 0.001
- Houkin	-7.000	-9.539	-4.461	1.296	< 0.001
- Laskowitz	-6.492	-9.118	-3.867	1.340	< 0.001
- Law	-6.068	-8.489	-3.646	1.235	< 0.001
- Prasad	-6.736	-9.765	-3.706	1.546	< 0.001

Figure S7: Leave one out analysis for the difference between the mean change in the NIHSS score.

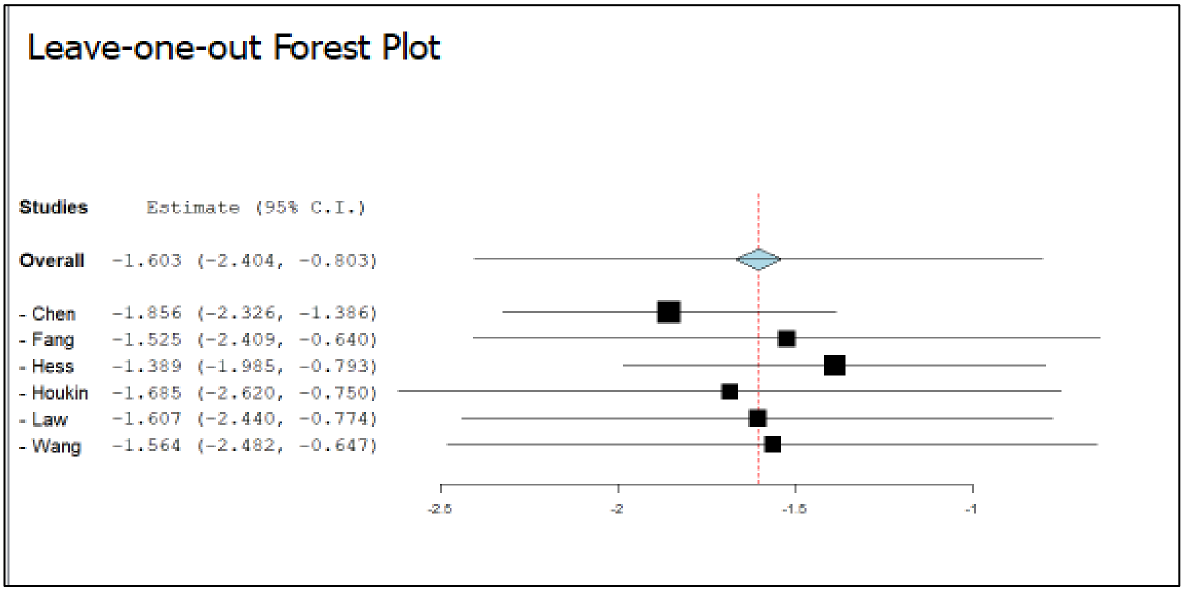

Studies	Estimate	Lower bound	Upper bound	Std. error	p-Val
Overall	-1.603	-2.404	-0.803	0.408	< 0.001
- Chen	-1.856	-2.326	-1.386	0.240	< 0.001
- Fang	-1.525	-2.409	-0.640	0.451	< 0.001
- Hess	-1.389	-1.985	-0.793	0.304	< 0.001
- Houkin	-1.685	-2.620	-0.750	0.477	< 0.001
- Law	-1.607	-2.440	-0.774	0.425	< 0.001
- Wang	-1.564	-2.482	-0.647	0.468	< 0.001

Figure S8: Leave one out analysis for the difference between the mean change in the mRS score.

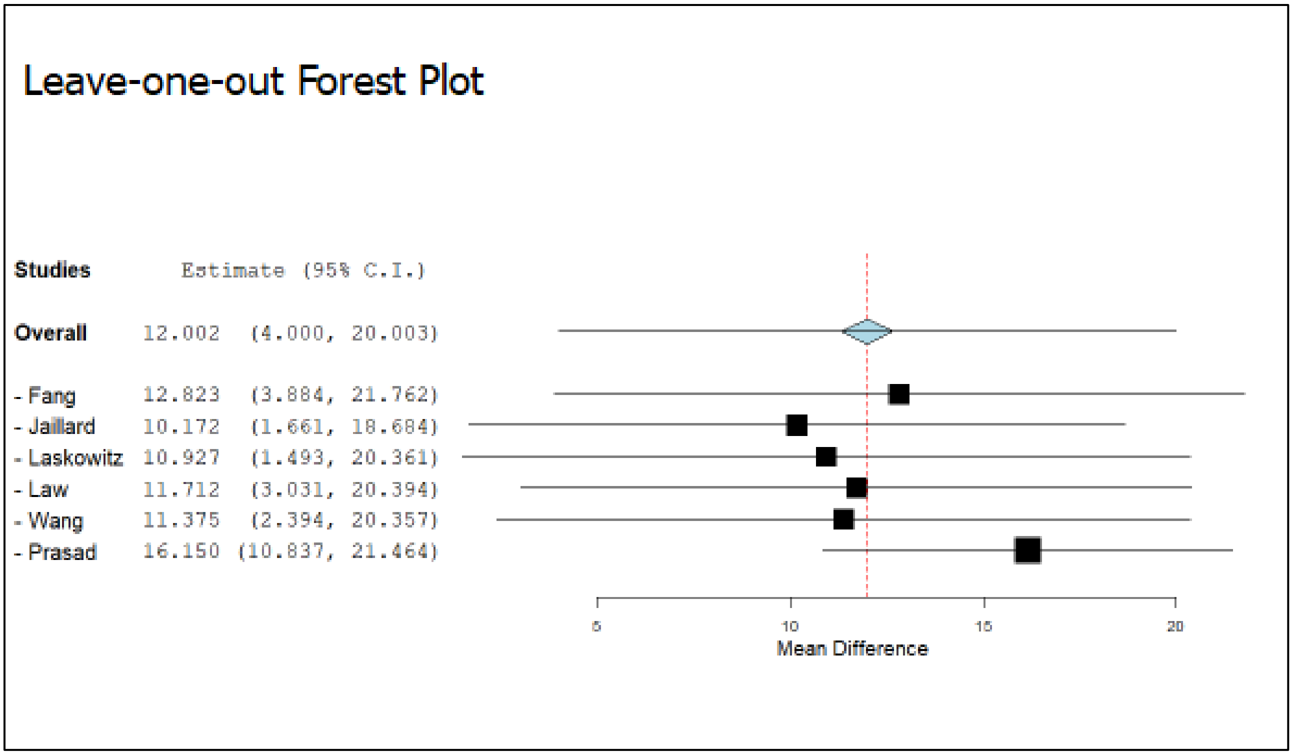
Leave-one-out Forest Plot
Studies
Estimate (95% C.I.)
Overall 12.002 (4.000, 20.003)
- Fang 12.823 (3.884, 21.762)
- Jaillard 10.172 (1.661, 18.684)
- Laskowitz 10.927 (1.493, 20.361)
- Law 11.712 (3.031, 20.394)
- Wang 11.375 (2.394, 20.357)
- Prasad 16.150 (10.837, 21.464)
5
10
15
20
Mean Difference

Studies	Estimate	Lower bound	Upper bound	Std. error	p-Val
Overall	12.002	4.000	20.003	4.083	0.003
- Fang	12.823	3.884	21.762	4.561	0.005
- Jaillard	10.172	1.661	18.684	4.343	0.019
- Laskowitz	10.927	1.493	20.361	4.813	0.023
- Law	11.712	3.031	20.394	4.430	0.008
- Wang	11.375	2.394	20.357	4.583	0.013
- Prasad	16.150	10.837	21.464	2.711	< 0.001

Figure S9: Leave one out analysis for the difference between the mean change in the BI.

Serious Adverse Events

In the SCT group, a total of 16 studies were analyzed, encompassing 482 subjects. The analysis, performed using a random effects model with the inverse variance method, estimated the summarized proportion of serious adverse events at 0.59 (95% CI: 0.55 - 0.63). A significant heterogeneity was detected ($p<0.01$), with an I^2 value of 92%, indicating that the variability among studies was primarily due to heterogeneity rather than random chance (Figure S10 A).

Similarly, in the placebo group, 15 studies with a total of 444 subjects were analyzed. The summarized proportion of serious adverse events in this group was 0.60 (95% CI: 0.56 - 0.64), based on the same statistical model. As with the SCT group, significant heterogeneity was observed ($p<0.01$), with an I^2 value of 92%, reflecting inconsistency in the magnitude or direction of effects across studies (Figure S10 B).

When comparing the SCT and placebo groups, the analysis demonstrated no statistically significant difference in the incidence of serious adverse events. The comparison, conducted using a random effects model with the Mantel-Haenszel method, yielded an overall risk ratio of 1.03 (95% CI: 0.87 - 1.23). The test for overall effect did not indicate a significant difference between the two groups. Moreover, no significant heterogeneity was observed, suggesting that effect sizes were relatively uniform across studies in both magnitude and direction (Figure S10 C).

A

Study	Events	Total	Weight	IV, Random, 95% CI
Bang, et al 2005	0	5	4.9%	0.00 [0.00; 0.52]
Bhasin et al 2015	0	20	6.4%	0.00 [0.00; 0.17]
V. Bhatia, et al 2018	1	10	5.8%	0.10 [0.00; 0.45]
Cherng Chen, et al 2014	0	15	6.2%	0.00 [0.00; 0.22]
Jie Fang, et al 2019	0	6	5.2%	0.00 [0.00; 0.46]
Yunliang Wang,et al 2020	0	9	5.7%	0.00 [0.00; 0.34]
Prasad 2014	12	60	6.9%	0.20 [0.11; 0.32]
LEE 2010	12	16	6.3%	0.75 [0.48; 0.93]
Savitz 2018	15	29	6.7%	0.52 [0.33; 0.71]
Chung 2021	0	39	6.8%	0.00 [0.00; 0.09]
Hess et al 2017	22	65	7.0%	0.34 [0.23; 0.47]
de Celis-Ruiz1 et al 2021	0	9	5.7%	0.00 [0.00; 0.34]
Moniche et al 2023	0	39	6.8%	0.00 [0.00; 0.09]
Zhe Kang Law et al 2021	0	9	5.7%	0.00 [0.00; 0.34]
Laskowitz et al 2023	1	47	6.9%	0.02 [0.00; 0.11]
Houkin 2024	55	104	7.1%	0.53 [0.43; 0.63]
**Total (95% CI)**		**482**	**100.0%**	**0.10 [0.01; 0.23]**
**Prediction interval**				**[0.00; 0.77]**

Heterogeneity: $Tau^2 = 0.0984$; $Chi^2 = 186.59$, df = 15 ($P < 0.01$); $I^2 = 92\%$

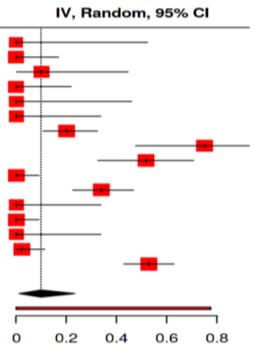

B

Study	Events	Total	Weight	IV, Random, 95% CI
Bhasin et al 2015	0	20	6.8%	0.00 [0.00; 0.17]
V. Bhatia, et al 2018	2	10	6.2%	0.20 [0.03; 0.56]
Cherng Chen, et al 2014	0	15	6.5%	0.00 [0.00; 0.22]
Jie Fang, et al 2019	0	6	5.5%	0.00 [0.00; 0.46]
Yunliang Wang,et al 2020	0	18	6.7%	0.00 [0.00; 0.19]
Prasad 2014	8	60	7.2%	0.13 [0.06; 0.25]
LEE 2010	29	36	7.1%	0.81 [0.64; 0.92]
Savitz 2018	10	19	6.7%	0.53 [0.29; 0.76]
Chung 2021	0	15	6.5%	0.00 [0.00; 0.22]
Hess et al 2017	24	61	7.3%	0.39 [0.27; 0.53]
de Celis-Ruiz1 et al 2021	4	10	6.2%	0.40 [0.12; 0.74]
Moniche et al 2023	0	38	7.1%	0.00 [0.00; 0.09]
Zhe Kang Law et al 2021	0	8	5.9%	0.00 [0.00; 0.37]
Laskowitz et al 2023	0	26	6.9%	0.00 [0.00; 0.13]
Houkin 2024	45	102	7.4%	0.44 [0.34; 0.54]
**Total (95% CI)**		**444**	**100.0%**	**0.13 [0.03; 0.29]**
**Prediction interval**				**[0.00; 0.84]**

Heterogeneity: $Tau^2 = 0.1076$; $Chi^2 = 185.97$, df = 14 ($P < 0.01$); $I^2 = 92\%$

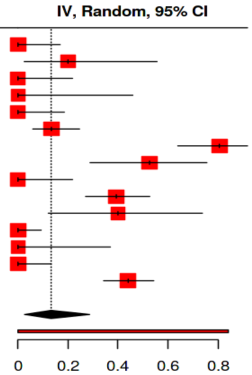

C

Study	Experimental Events	Experimental Total	Control Events	Control Total	Weight	Risk Ratio MH, Random, 95% CI
Bhasin et al 2015	0	20	0	20	0.0%	
V. Bhatia, et al 2018	1	10	2	10	0.6%	0.50 [0.05; 4.67]
Cherng Chen, et al 2014	0	15	0	15	0.0%	
Jie Fang, et al 2019	0	6	0	6	0.0%	
Yunliang Wang,et al 2020	0	9	0	18	0.0%	
Prasad 2014	12	60	8	60	4.7%	1.50 [0.66; 3.41]
LEE 2010	12	16	29	36	29.8%	0.93 [0.67; 1.29]
Savitz 2018	15	29	10	19	10.3%	0.98 [0.57; 1.71]
Chung 2021	0	39	0	15	0.0%	
Hess et al 2017	22	65	24	61	14.8%	0.86 [0.54; 1.36]
de Celis-Ruiz1 et al 2021	0	9	4	10	0.4%	0.12 [0.01; 1.99]
Moniche et al 2023	0	39	0	38	0.0%	
Zhe Kang Law et al 2021	0	9	0	8	0.0%	
Laskowitz et al 2023	1	47	0	26	0.3%	1.67 [0.07; 39.65]
Houkin 2024	55	104	45	102	39.1%	1.20 [0.90; 1.59]
**Total (95% CI)**		**477**		**444**	**100.0%**	**1.03 [0.87; 1.23]**
**Prediction interval**						**[0.83; 1.29]**

Heterogeneity: $Tau^2 = 0$; $Chi^2 = 5.62$, df = 7 ($P = 0.59$); $I^2 = 0\%$
Test for overall effect: $Z = 0.37$ ($P = 0.71$)

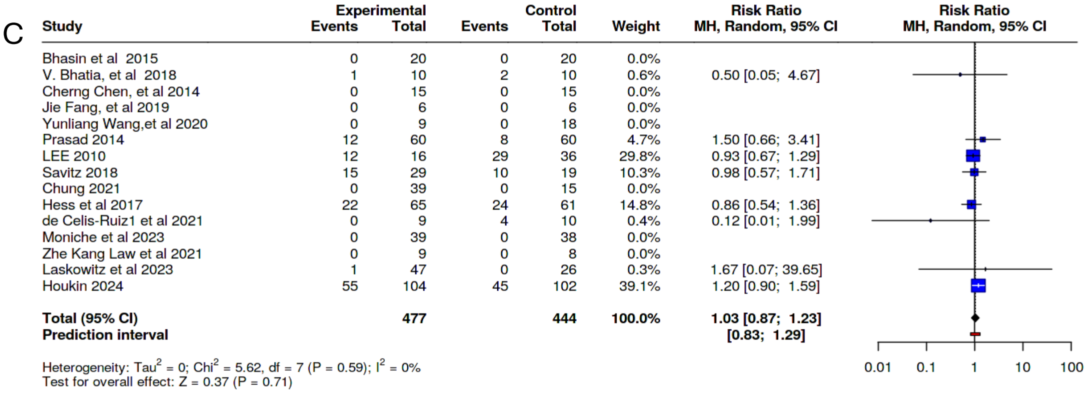

Figure S10. Forest plot of the incidence of serious adverse events in A) SCT group and B) placebo group and C) difference between SCT and control group.

Immediate Adverse Events

A total of 16 studies involving 394 subjects were analyzed to assess the incidence of immediate adverse events in the SCT group. The summarized proportion of these events was estimated at 0.56 (95% CI: 0.50 - 0.63) using a random effects model with the inverse variance method. A significant heterogeneity was detected ($p<0.01$), with an I^2 value of 96%, indicating substantial variability in the reported rates across studies (Figure S11 A).

In the placebo group, 15 studies comprising 357 subjects were analyzed. The estimated proportion of immediate adverse events was 0.57 (95% CI: 0.50 - 0.63), with similarly high heterogeneity ($p<0.01$, $I^2=96\%$). This suggests that study-specific factors may have contributed to the variability in reported event rates (Figure S11 B).

A direct comparison between the SCT and placebo groups was conducted using data from three studies involving 389 subjects in the SCT group and 357 in the placebo group. The analysis, performed using a random effects model with the Mantel-Haenszel method, yielded a risk ratio of 0.99 (95% CI: 0.51 - 1.94), indicating no significant difference between the two cohorts. The test for overall effect did not show a statistically significant effect, and a moderate level of heterogeneity was detected ($p=0.06$, $I^2=65\%$), suggesting some inconsistency in the effect sizes across studies (Figure S11 C).

A

Study	Events	Total	Weight	IV, Random, 95% CI
Bang, et al 2005	0	5	5.7%	0.00 [0.00; 0.52]
Bhasin et al 2015	0	20	6.4%	0.00 [0.00; 0.17]
Assia Jaillard, et al 2020	0	16	6.3%	0.00 [0.00; 0.21]
V. Bhatia, et al 2018	0	10	6.1%	0.00 [0.00; 0.31]
Cherng Chen, et al 2014	0	15	6.3%	0.00 [0.00; 0.22]
Jie Fang, et al 2019	0	6	5.8%	0.00 [0.00; 0.46]
Yunliang Wang,et al 2020	0	9	6.0%	0.00 [0.00; 0.34]
Prasad 2014	0	60	6.6%	0.00 [0.00; 0.06]
LEE 2010	8	16	6.3%	0.50 [0.25; 0.75]
Savitz 2018	0	29	6.4%	0.00 [0.00; 0.12]
Chung 2021	0	39	6.5%	0.00 [0.00; 0.09]
Hess et al 2017	64	65	6.6%	0.98 [0.92; 1.00]
de Celis-Ruiz1 et al 2021	0	9	6.0%	0.00 [0.00; 0.34]
Moniche et al 2023	0	39	6.5%	0.00 [0.00; 0.09]
Zhe Kang Law et al 2021	0	9	6.0%	0.00 [0.00; 0.34]
Laskowitz et al 2023	0	47	6.5%	0.00 [0.00; 0.08]
**Total (95% CI)**		**394**	**100.0%**	**0.03 [0.00; 0.22]**
**Prediction interval**				**[0.00; 0.99]**

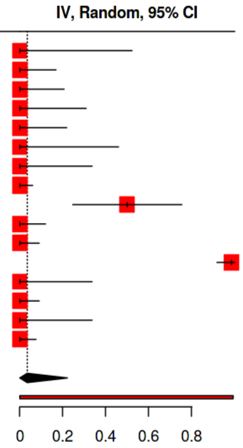

Heterogeneity: $Tau^2 = 0.2632$; $Chi^2 = 396.68$, df = 15 ($P < 0.01$); $I^2 = 96\%$

B

Study	Events	Total	Weight	IV, Random, 95% CI
Bhasin et al 2015	0	20	6.7%	0.00 [0.00; 0.17]
Assia Jaillard, et al 2020	0	15	6.6%	0.00 [0.00; 0.22]
V. Bhatia, et al 2018	0	10	6.4%	0.00 [0.00; 0.31]
Cherng Chen, et al 2014	0	15	6.6%	0.00 [0.00; 0.22]
Jie Fang, et al 2019	0	6	6.1%	0.00 [0.00; 0.46]
Yunliang Wang,et al 2020	0	18	6.7%	0.00 [0.00; 0.19]
Prasad 2014	0	60	7.0%	0.00 [0.00; 0.06]
LEE 2010	13	36	6.9%	0.36 [0.21; 0.54]
Savitz 2018	0	19	6.7%	0.00 [0.00; 0.18]
Chung 2021	0	15	6.6%	0.00 [0.00; 0.22]
Hess et al 2017	59	61	7.0%	0.97 [0.89; 1.00]
de Celis-Ruiz1 et al 2021	0	10	6.4%	0.00 [0.00; 0.31]
Moniche et al 2023	0	38	6.9%	0.00 [0.00; 0.09]
Zhe Kang Law et al 2021	0	8	6.3%	0.00 [0.00; 0.37]
Laskowitz et al 2023	4	26	6.8%	0.15 [0.04; 0.35]
**Total (95% CI)**		**357**	**100.0%**	**0.05 [0.00; 0.23]**
**Prediction interval**				**[0.00; 0.98]**

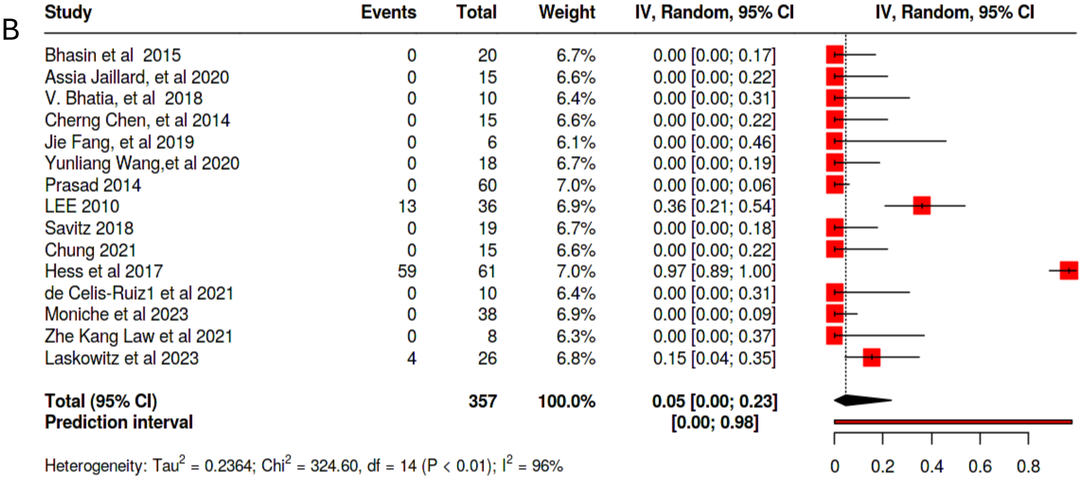

Heterogeneity: $Tau^2 = 0.2364$; $Chi^2 = 324.60$, df = 14 ($P < 0.01$); $I^2 = 96\%$

Study	Experimental Events	Experimental Total	Control Events	Control Total	Weight	Risk Ratio MH, Random, 95% CI
Bhasin et al 2015	0	20	0	20	0.0%	
Assia Jaillard, et al 2020	0	16	0	15	0.0%	
V. Bhatia, et al 2018	0	10	0	10	0.0%	
Cherng Chen, et al 2014	0	15	0	15	0.0%	
Jie Fang, et al 2019	0	6	0	6	0.0%	
Yunliang Wang,et al 2020	0	9	0	18	0.0%	
Prasad 2014	0	60	0	60	0.0%	
LEE 2010	8	16	13	36	37.3%	1.38 [0.72; 2.67]
Savitz 2018	0	29	0	19	0.0%	
Chung 2021	0	39	0	15	0.0%	
Hess et al 2017	64	65	59	61	57.7%	1.02 [0.96; 1.08]
de Celis-Ruiz1 et al 2021	0	9	0	10	0.0%	
Moniche et al 2023	0	39	0	38	0.0%	
Zhe Kang Law et al 2021	0	9	0	8	0.0%	
Laskowitz et al 2023	0	47	4	26	5.0%	0.06 [0.00; 1.11]
**Total (95% CI)**		**389**		**357**	**100.0%**	**0.99 [0.51; 1.94]**
**Prediction interval**						**[0.00; 1310.22]**

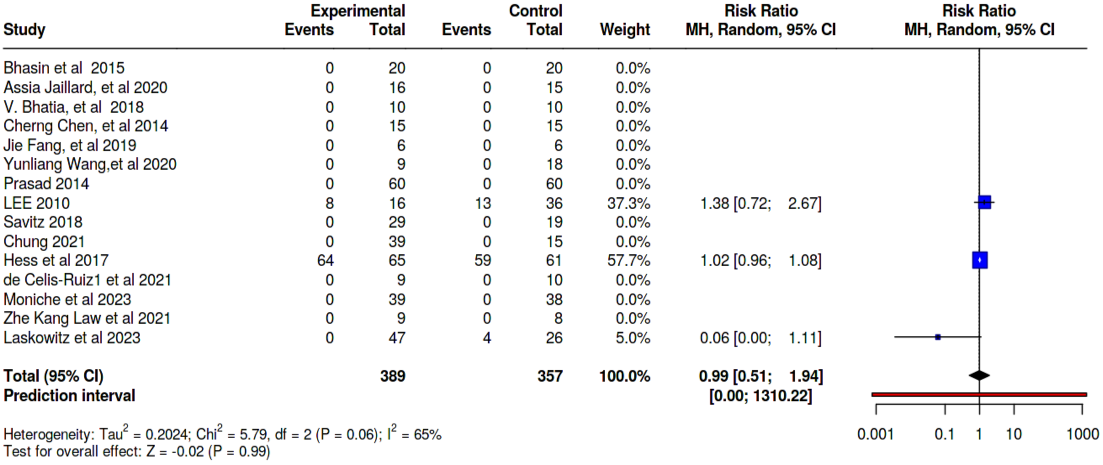

Heterogeneity: $Tau^2 = 0.2024$; $Chi^2 = 5.79$, df = 2 ($P = 0.06$); $I^2 = 65\%$
Test for overall effect: Z = -0.02 (P = 0.99)

Figure S11. Forest plot of the incidence of immediate adverse events in A) SCT and B) placebo groups and C) The difference between SCT and placebo groups.

Delayed Adverse Events

In the SCT group, 15 studies comprising 453 subjects were analyzed to assess the prevalence of delayed adverse events. The summarized proportion, based on a random effects model with the inverse variance method, was estimated at 0.58 (95% CI: 0.54 - 0.63). A significant heterogeneity was detected ($p<0.01$), with an I^2 value of 93%, indicating that the majority of variability among studies was due to heterogeneity rather than random variation (Figure S12 A).

Similarly, in the placebo group, 14 studies with a total of 425 subjects were analyzed. The estimated proportion of delayed adverse events was 0.59 (95% CI: 0.55 - 0.63), with significant heterogeneity observed ($p<0.01$, $I^2=93\%$). These findings suggest that delayed adverse events were similarly prevalent in both the SCT and placebo groups, with substantial variability across studies (Figure S12 B).

When comparing the SCT and placebo groups, six studies involving 369 subjects in the experimental cohort and 361 in the control cohort were analyzed. The comparison, conducted using a random effects model with the Mantel-Haenszel method, revealed no significant difference between the two cohorts, with an overall risk ratio of 0.97 (95% CI: 0.66 - 1.40). The test for overall effect indicate some difference, and a moderate level of heterogeneity was detected ($p=0.02$, $I^2=64\%$), suggesting some inconsistency in reported effect sizes across studies (Figure S12 C).

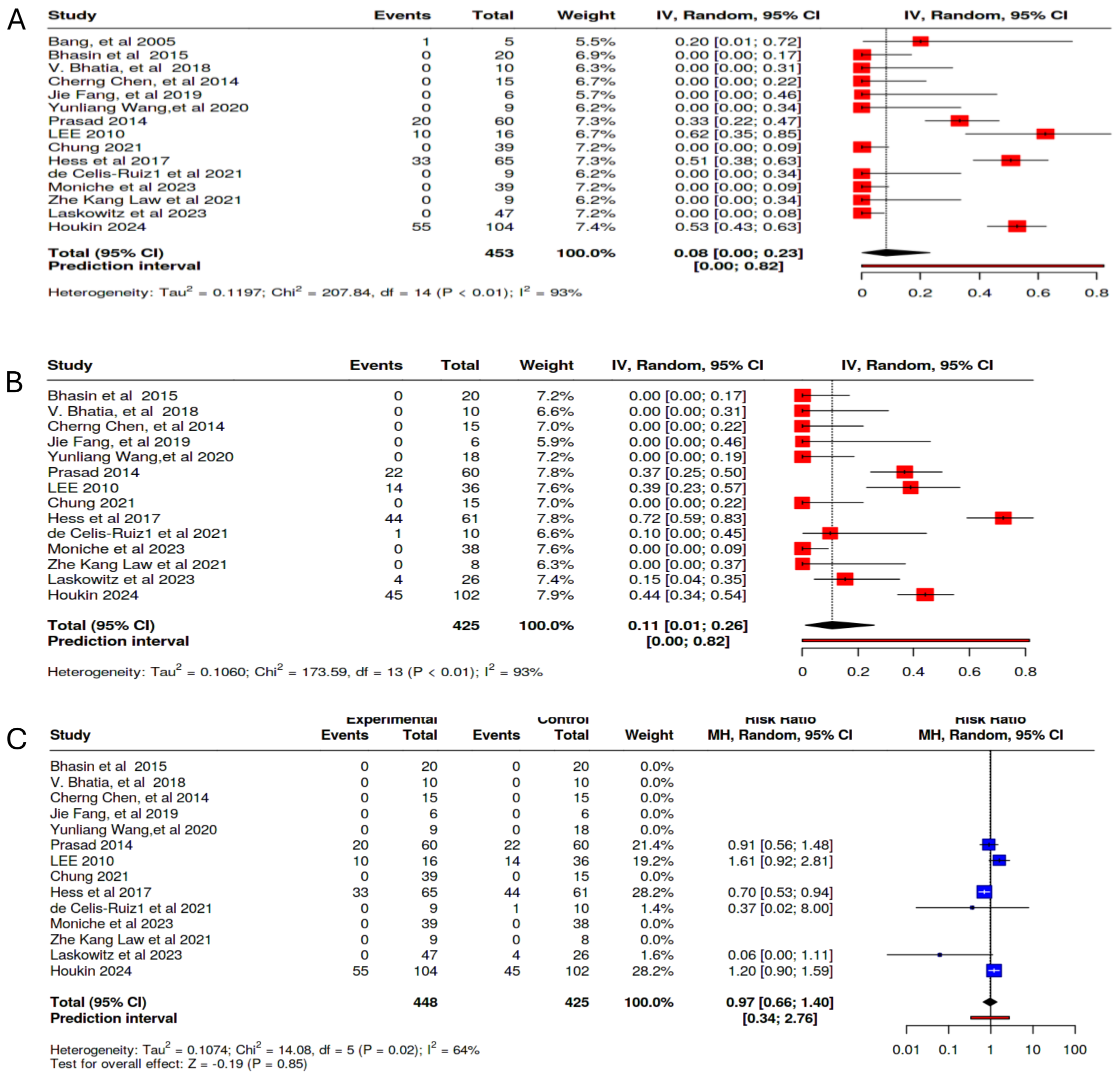

Figure S12. Forest plot of the incidence of delayed adverse events in a) SCT and B) placebo groups and C) Difference between SCT and placebo group.

Subgroup analysis

Different subgroup analyses were performed to explore the observed heterogeneity for the primary outcomes of difference in the mean change of NIHSS score, mRS score, and BI. Subgroup analyses for the difference in the mean change of NIHSS score were performed based on the region for conducting the original study (USA, European or Asian countries), age (<60 years old or >60 years old), patients count of the pooled studies (n <40 or n >40) and gender distribution of the studies (males <70% or >70%). Sub-group analyses demonstrated that the decrease in the mean change of the NIHSS score in stem cell therapy patients was greater than that in the controls in the studies conducted in Asian countries (Figure S13)

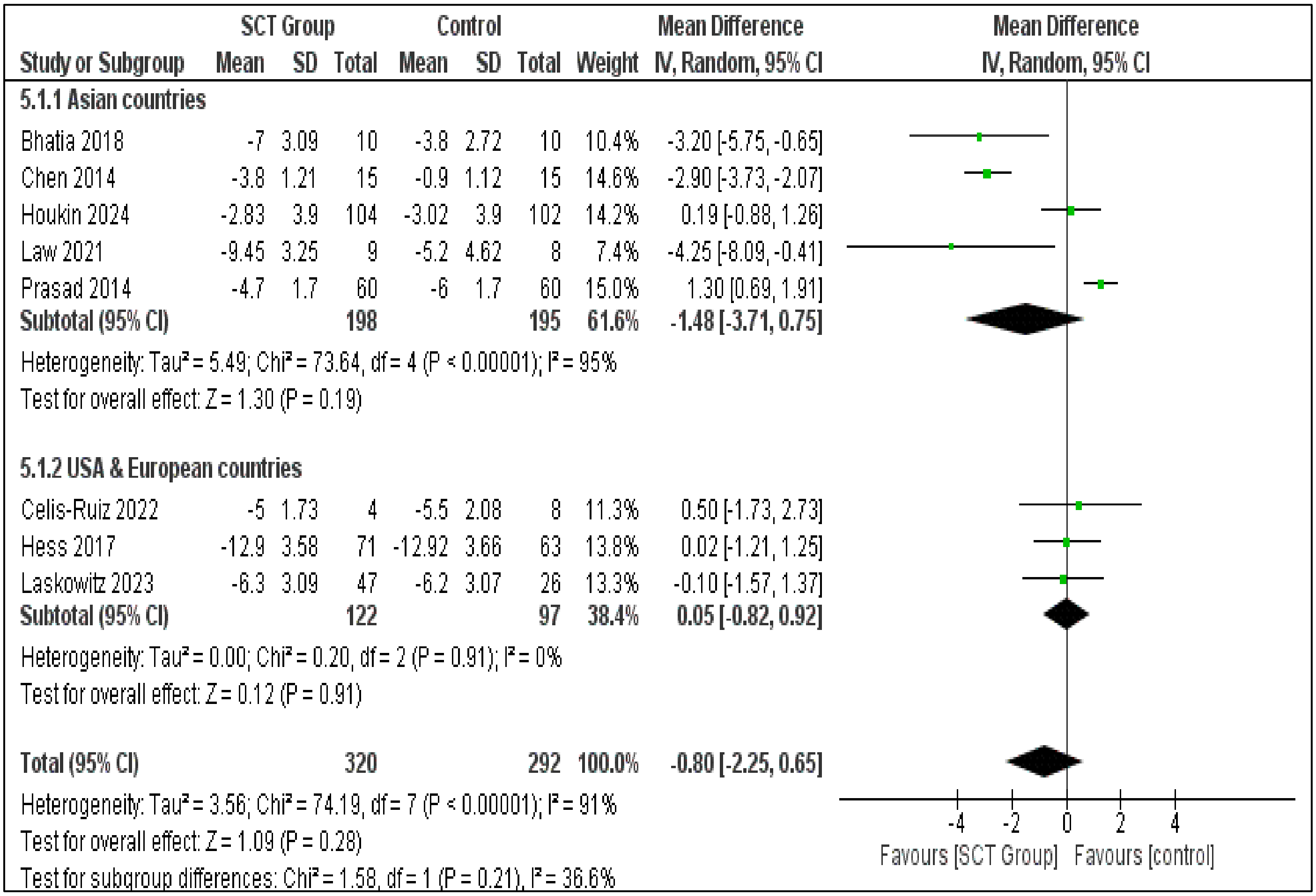

Figure S13: Sub-grouping of the difference between the mean change in the NIHSS score by the countries of the original papers.

Sub-group analyses demonstrated that the decrease in the mean change of NIHSS score in the stem cell therapy patients was greater than in the controls in the studies with a mean age <60 years (Figure S14).

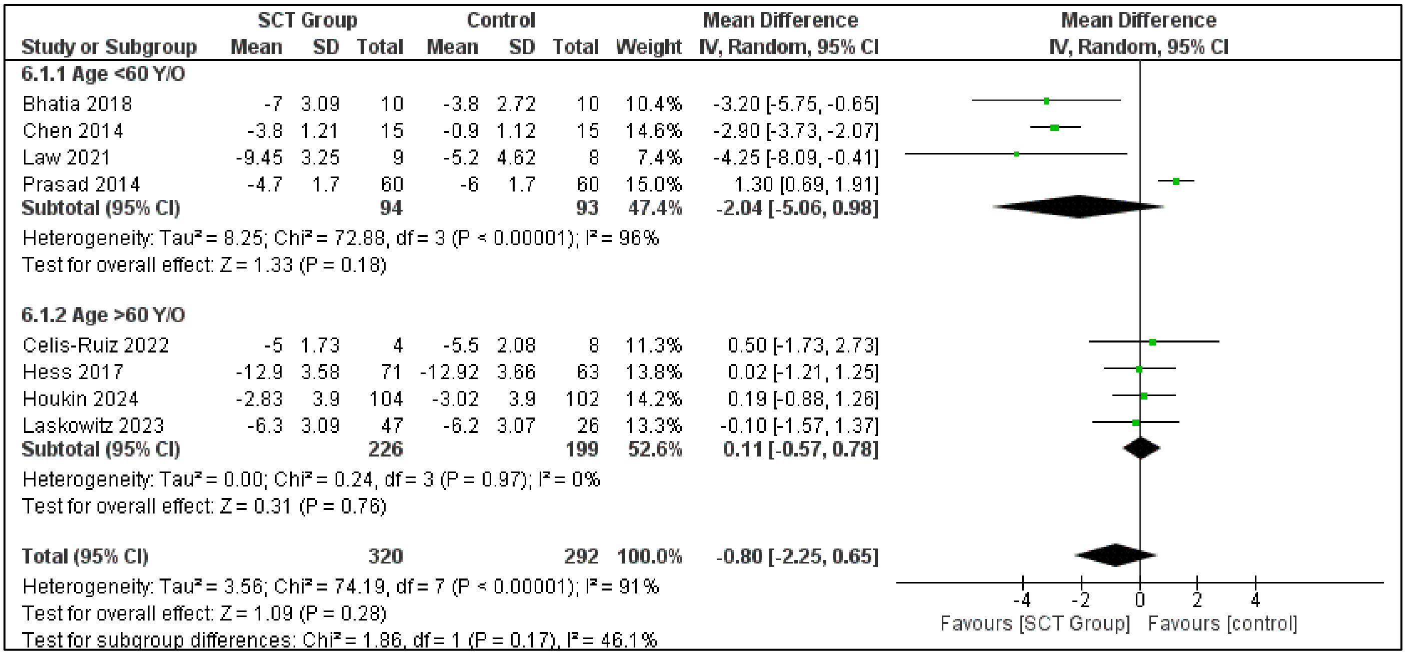

Figure S14: Sub-grouping of the difference between the mean change in the NIHSS score by the age distribution of the studied population.

Sub-group analyses demonstrated that the decrease in the mean change of NIHSS score in the stem cell therapy patients was greater than in the controls in the studies with the patients n<40 (Figure S15).

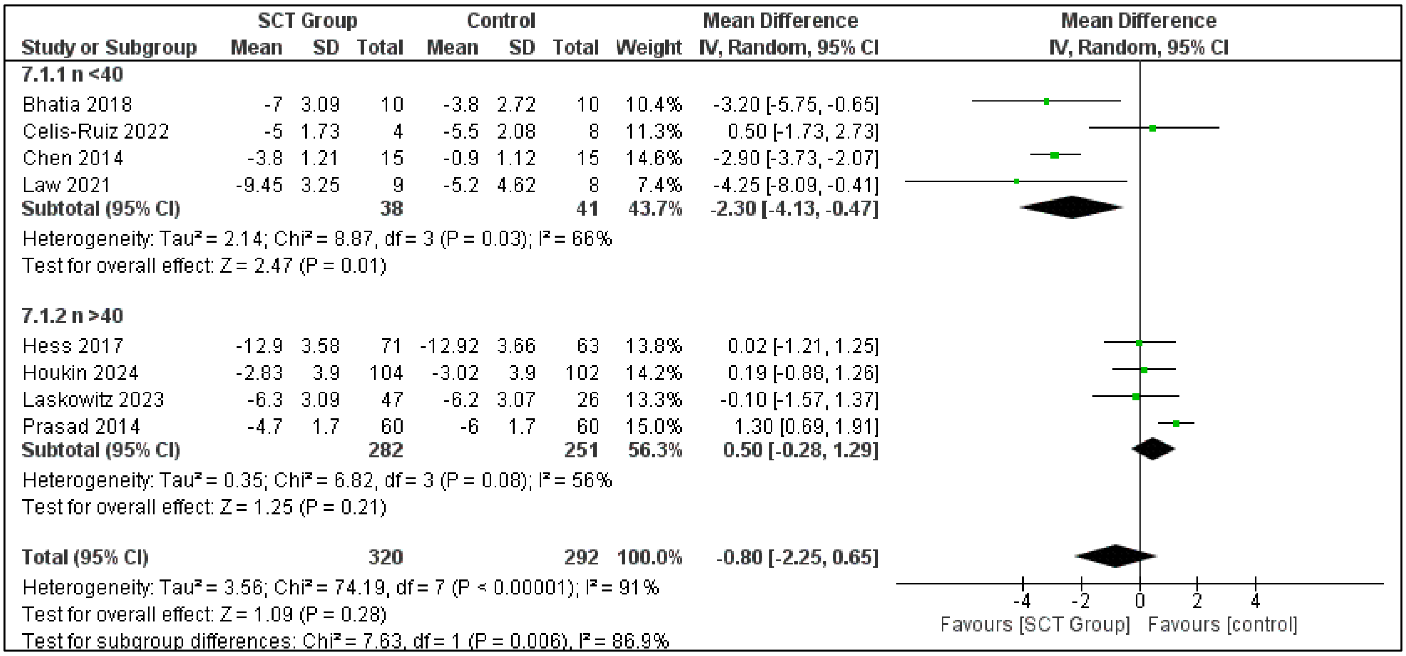

Figure S15: Sub-grouping of the difference between the mean change in the NIHSS score by the patient count.

Sub-group analyses demonstrated that the decrease in the mean change of the NIHSS score in the stem cell therapy patients was greater than in the controls in the studies with males >70% of the study population (Figure S16).

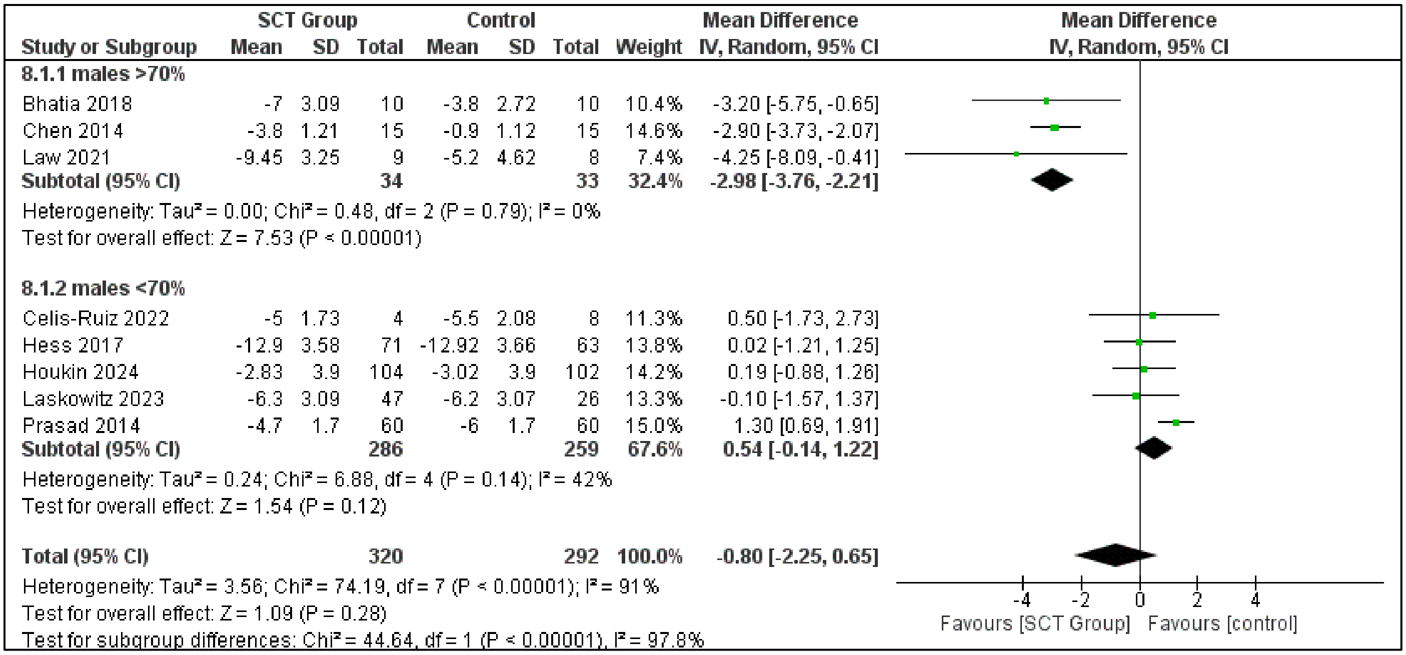

Figure S16: Sub-grouping of the difference between the mean change in the NIHSS score by the gender distribution in the studies.

Subgroup analyses for the difference in the mean change of mRS score were performed based on the duration of stroke in the patients of the original study (hours-to-days or months-to-years), patients count of the pooled studies (n <50 or n >50) and gender distribution of the studies (males <70% or >70%). Sub-group analyses demonstrated that the decrease in the mean change of mRS score in the stem cell therapy patients was greater as compared to the controls in the studies with the duration of stroke in months to years (Figure S17).

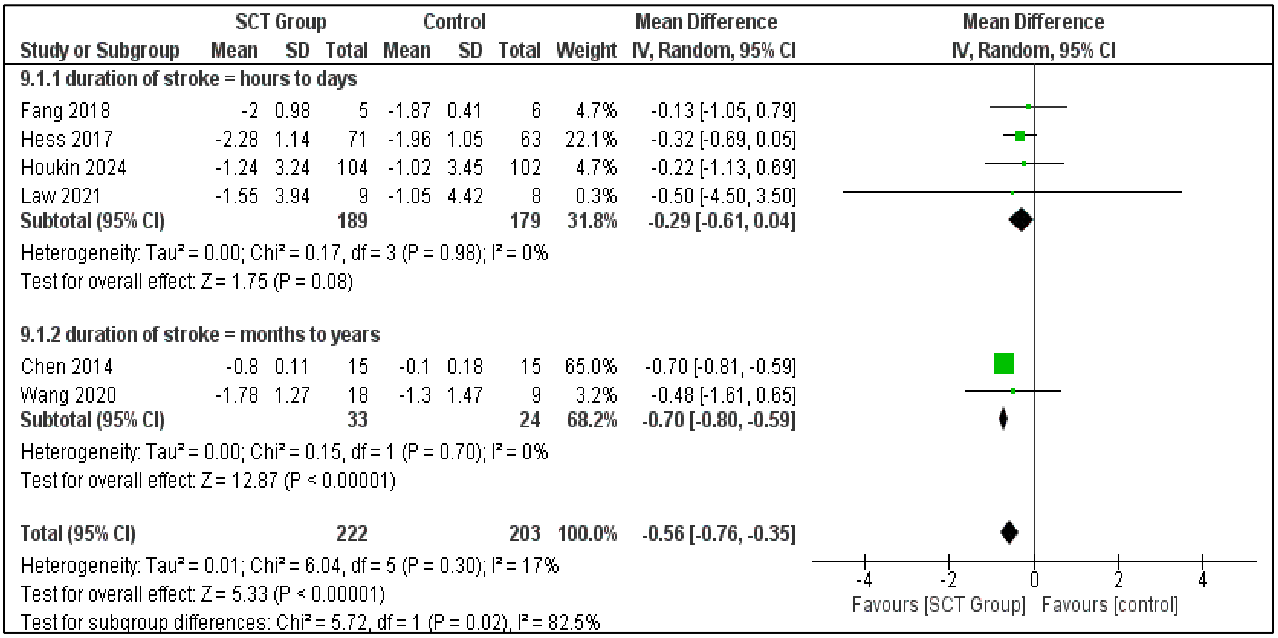

Figure S17: Sub-grouping of the difference between the mean change in the mRS score by the duration of stroke in the original papers.

Sub-group analyses demonstrated that the decrease in the mean change of mRS score in the stem cell therapy patients was greater as compared to the controls in the studies with the patients n<50 (Figure S18).

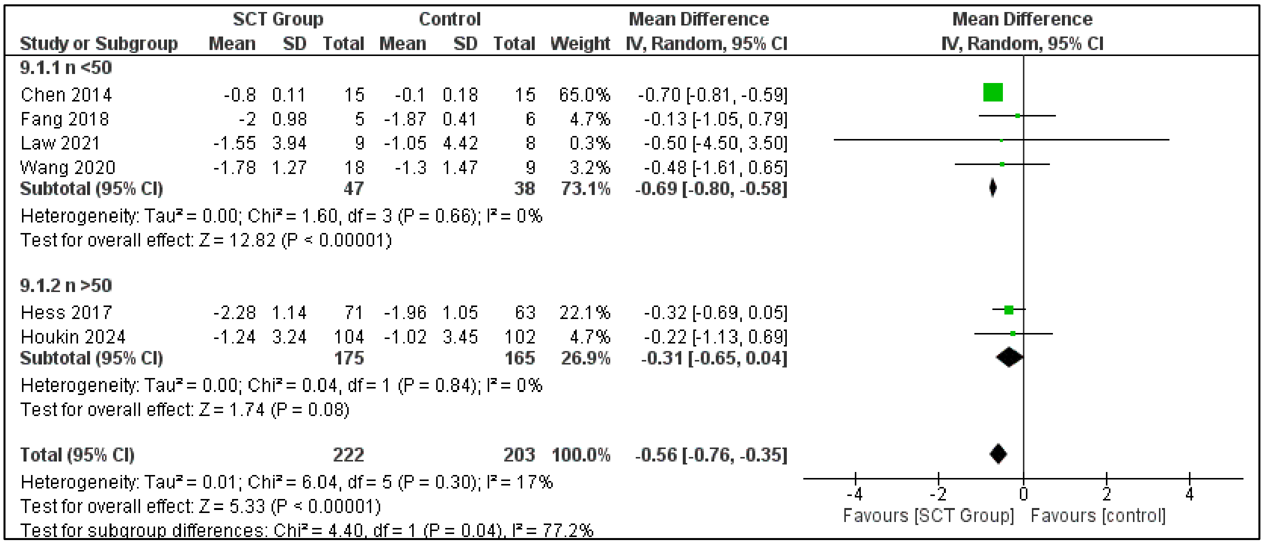

Figure S18: Sub-grouping of the difference between the mean change in the mRS score by the patient count of the original papers.

Sub-group analyses demonstrated that the decrease in the mean change of mRS score in the stem cell therapy patients was greater as compared to the controls in the studies with the males >70% of the study population (Figure S19).

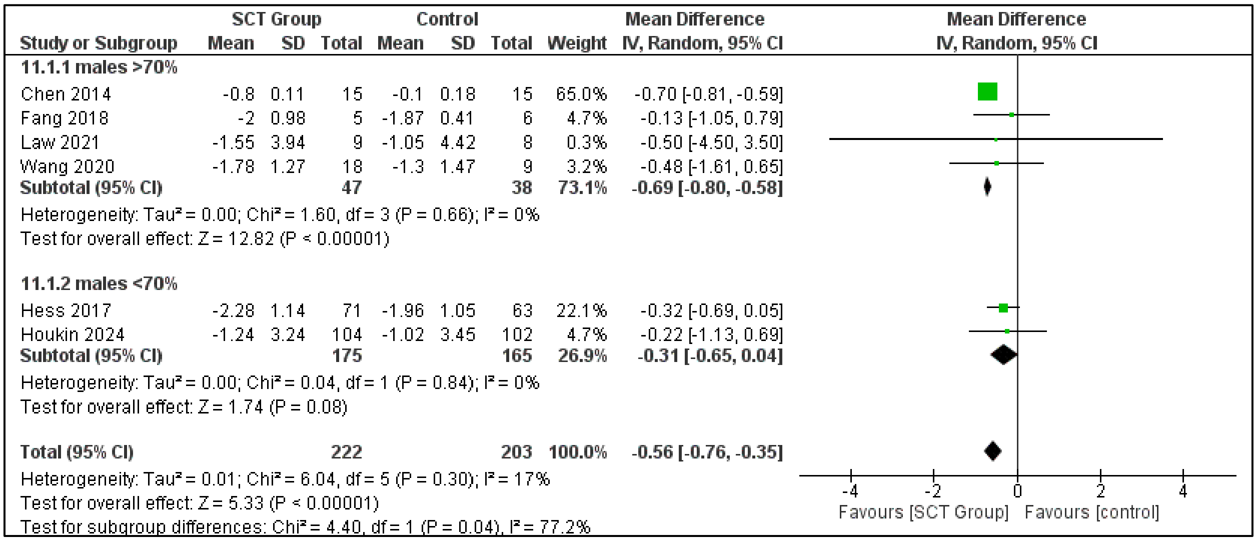

Figure S19: Sub-grouping of the difference between the mean change in the mRS score by the patient's gender distribution of the original papers.

Subgroup analyses for the difference in the mean change of BI were performed based on the region for conducting the original study (USA, France or Asian countries), the duration of stroke in the patients of the original study (<2 months

or >2 months), duration of follow-up of the patients (12 months and less or more than 12 months) and gender distribution of the studies (males <70% or >70%).

Sub-group analyses demonstrated that the increase in the mean change of Barthel index in the stem cell therapy patients was greater than in the controls in the studies conducted in the USA and France, as shown in Figure S20. Studies Fang et al. 2018 (23) and Jaillard et al. (2020) (34) were the sources of heterogeneity here.

	SCT Group			Control				Mean Difference
**Study or Subgroup**	**Mean**	**SD**	**Total**	**Mean**	**SD**	**Total**	**Weight**	**IV, Random, 95% CI**
**12.1.1 Asian countries**								
Fang 2018	55	18.63	5	49	13.59	6	10.5%	6.00 [-13.62, 25.62]
Law 2021	77	15.45	9	61.35	30.36	8	8.3%	15.65 [-7.68, 38.98]
Prasad 2014	38.1	13.75	60	36.1	13.65	60	25.6%	2.00 [-2.90, 6.90]
Wang 2020	37.85	19.16	18	21.6	19.53	9	13.7%	16.25 [0.72, 31.78]
**Subtotal (95% CI)**			**92**			**83**	**58.1%**	**6.30 [-1.16, 13.75]**
Heterogeneity: Tau² = 17.61; Chi² = 4.02, df = 3 (P = 0.26); I² = 25%								
Test for overall effect: Z = 1.66 (P = 0.10)								
**12.1.2 USA & France**								
Jaillard 2020	36.26	19.13	16	17	8.29	15	19.3%	19.26 [8.99, 29.53]
Laskowitz 2023	38	15.18	47	22	16.2	26	22.6%	16.00 [8.41, 23.59]
**Subtotal (95% CI)**			**63**			**41**	**41.9%**	**17.15 [11.05, 23.26]**
Heterogeneity: Tau² = 0.00; Chi² = 0.25, df = 1 (P = 0.62); I² = 0%								
Test for overall effect: Z = 5.51 (P < 0.00001)								
**Total (95% CI)**			**155**			**124**	**100.0%**	**12.00 [4.00, 20.00]**
Heterogeneity: Tau² = 58.83; Chi² = 16.10, df = 5 (P = 0.007); I² = 69%								
Test for overall effect: Z = 2.94 (P = 0.003)								
Test for subgroup differences: Chi² = 4.88, df = 1 (P = 0.03), I² = 79.5%								

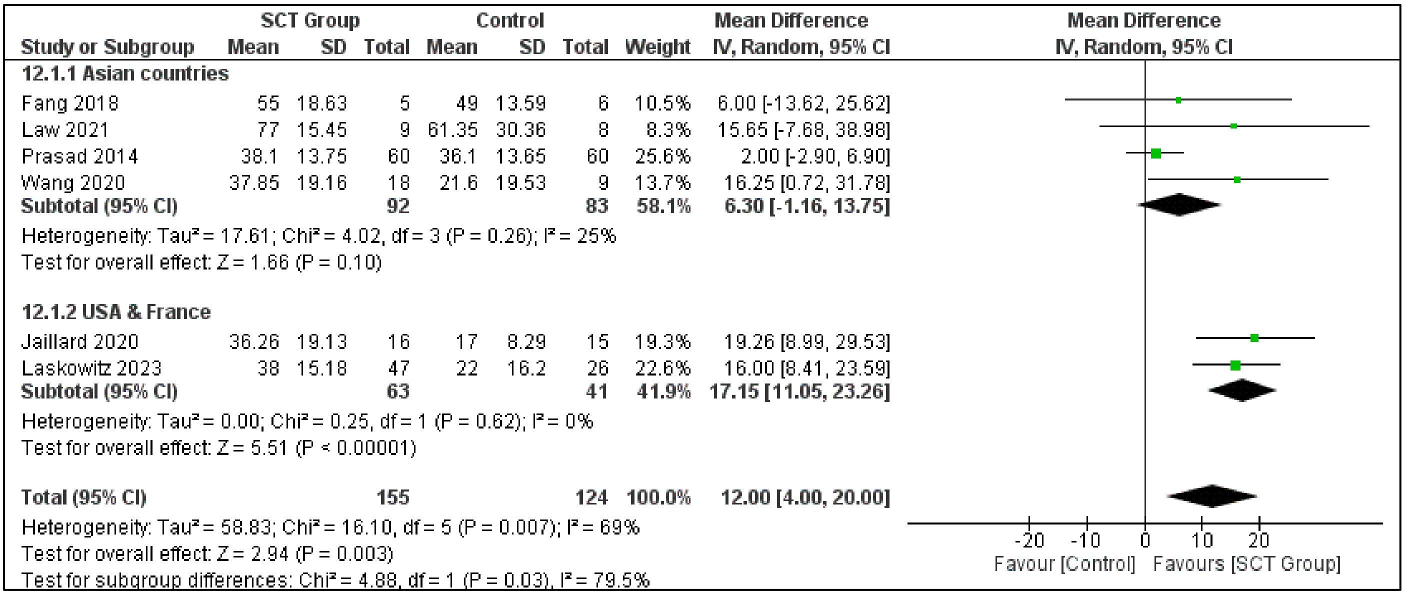

Figure S20: Sub-grouping of the difference between the mean change in the Barthel index by the patient's country distribution of the original papers.

Sub-group analyses demonstrated that the increase in the mean change of BI in the stem cell therapy patients was greater as compared to the controls in the studies with males <70% of the study population (Figure S21).

	SCT Group			Control				Mean Difference
**Study or Subgroup**	**Mean**	**SD**	**Total**	**Mean**	**SD**	**Total**	**Weight**	**IV, Random, 95% CI**
**13.1.1 males >70%**								
Fang 2018	55	18.63	5	49	13.59	6	10.5%	6.00 [-13.62, 25.62]
Law 2021	77	15.45	9	61.35	30.36	8	8.3%	15.65 [-7.68, 38.98]
Wang 2020	37.85	19.16	18	21.6	19.53	9	13.7%	16.25 [0.72, 31.78]
**Subtotal (95% CI)**			**32**			**23**	**32.5%**	**13.02 [2.22, 23.81]**
Heterogeneity: Tau² = 0.00; Chi² = 0.71, df = 2 (P = 0.70); I² = 0%								
Test for overall effect: Z = 2.36 (P = 0.02)								
**13.1.2 males <70%**								
Jaillard 2020	36.26	19.13	16	17	8.29	15	19.3%	19.26 [8.99, 29.53]
Laskowitz 2023	38	15.18	47	22	16.2	26	22.6%	16.00 [8.41, 23.59]
Prasad 2014	38.1	13.75	60	36.1	13.65	60	25.6%	2.00 [-2.90, 6.90]
**Subtotal (95% CI)**			**123**			**101**	**67.5%**	**11.85 [0.34, 23.35]**
Heterogeneity: Tau² = 87.88; Chi² = 14.64, df = 2 (P = 0.0007); I² = 86%								
Test for overall effect: Z = 2.02 (P = 0.04)								
**Total (95% CI)**			**155**			**124**	**100.0%**	**12.00 [4.00, 20.00]**
Heterogeneity: Tau² = 58.83; Chi² = 16.10, df = 5 (P = 0.007); I² = 69%								
Test for overall effect: Z = 2.94 (P = 0.003)								
Test for subgroup differences: Chi² = 0.02, df = 1 (P = 0.88), I² = 0%								

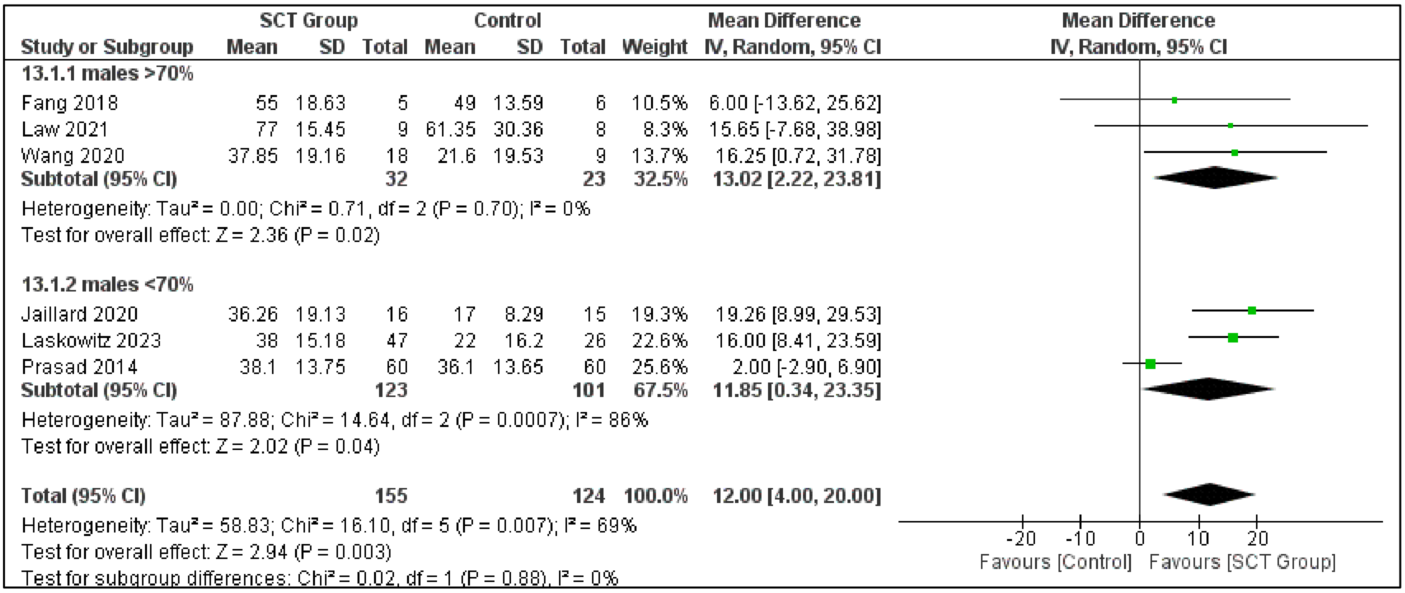

Figure S21: Sub-grouping of the difference between the mean change in the Barthel index by the patient's gender distribution of the original papers.

Sub-group analyses demonstrated that the mean change of BI in the stem cell therapy patients was similar as compared to the controls in the studies when grouped based on duration of stroke (Figure S22).

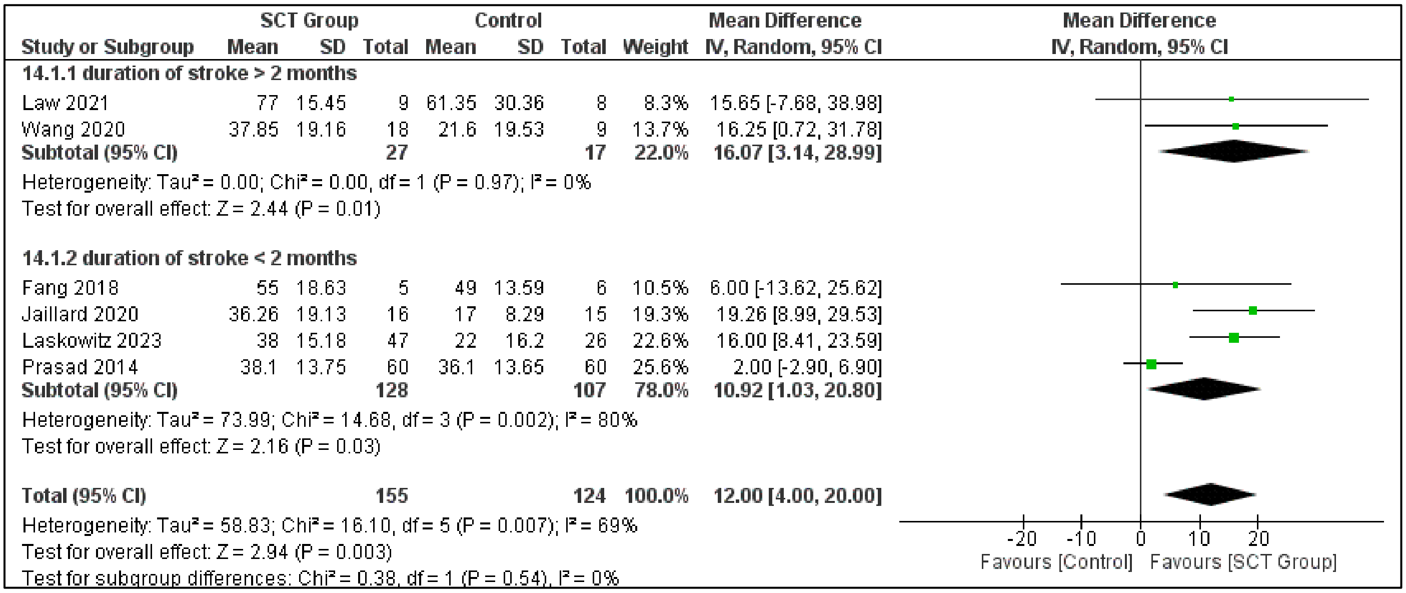

Figure S22: Sub-grouping of the difference between the mean change in the Barthel index by the duration of stroke in the original papers.

Sub-group analyses demonstrated that the mean change of BI in the stem cell therapy patients was similar as compared to the controls in the studies when grouped based on duration of follow-up (Figure S23).

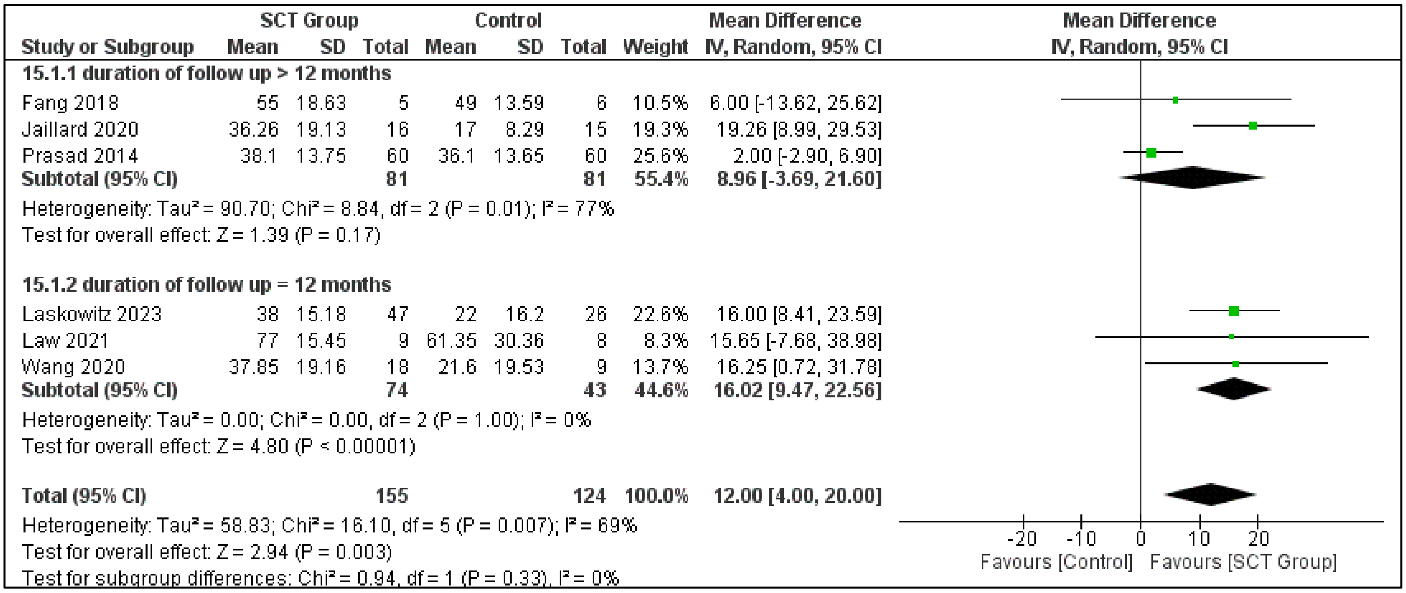

Figure S23: Sub-grouping of the difference between the mean change in the Barthel index by the duration of follow-up in the original papers.
